# Etiology, prevalence, and mortality of sepsis among children under five years in Africa: a systematic review and meta-analysis

**DOI:** 10.1186/s12879-026-13350-2

**Published:** 2026-04-25

**Authors:** Cheickna Hamallah Dicko, Deprince Nakensy N’Dri, Malak Snoussi, Néhémie Nzoyikorera, Abdou Azaque Zouré, Dinanibè Kambiré, Insaf Elammari, Amal Bouziyane, Mohamed Kettani Halabi, Berthold Bivigou-Mboumba, Kaotar Nayme, Berthe Amélie Iroungou, Mohamed Khalis, Assiya El Kettani, Mariam Sylla, Luria Leslie Founou, Abdelhamid Naitlho, Ouissal Aissaoui, Jalila El Bakkouri, Ali Jafri, Fatoumata Kontao, Alexander-Enoch-Nii-Martey Dickson, Naima Elmdaghri, Abdelfattah Chakib, Aziz Bousfiha, Mohammed Bouskraoui, Mohamed Adnaoui, Nouzha Dini, Ibrehima Guindo, Idrissa Diawara

**Affiliations:** 1 Research Laboratory of Microbiology, Infectious Diseases, Allergology and Pathogen Surveillance (LARMIAS), Mohammed VI Faculty of Medicine, Mohammed VI University of Sciences and Health (UM6SS), Casablanca, 82403 Morocco; 2 Mohammed VI Higher Institute of Biosciences and Biotechnologies, Mohammed VI University of Sciences and Health (UM6SS), Casablanca, 82403 Morocco; 3 National Public Health Institute, Hippodrome, Koulikoro Road – Street 235, Gate 52 – Commune II, Bamako, BP1771 Mali; 4 Interdisciplinary Laboratory of Biotechnology and Health, Mohammed VI Higher Institute of Biosciences and Biotechnology, Casablanca, 20190 Morocco; 5 Mohammed VI University of Sciences and Health (UM6SS), Casablanca, 20180 Morocco; 6 Cheikh Khalifa International University Hospital, Casablanca, 82403 Morocco; 7 National Reference Laboratory, National Public Health Institute, Bujumbura, Burundi; 8 Laboratory of Molecular and Genetic Biology (LABIOGENE), Joseph Ki-Zerbo University, Ouagadougou, Burkina Faso; 9 Pietro Annigoni Biomolecular Research Centre (CERBA), Ouagadougou, Burkina Faso; 10 Biomedical Research Laboratory (LaReBio), Biomedical and Public Health Department, Health Sciences Research Institute/National Center for Scientific and Technological Research (IRSS/CNRST), Ouagadougou, Burkina Faso; 11 Health Sciences Research Institute (IRSS), National Center for Scientific and Technological Research (CNRST), Ouagadougou, Burkina Faso; 12 Mohammed VI International University Hospital, Bouskoura, 27182 Morocco; 13 Research Laboratory in Drug Sciences, Mohammed VI Faculty of Pharmacy, Mohammed VI University of Sciences and Health, Casablanca, 82403 Morocco; 14 Unité Mixte de Recherches CIRMF-SSM (UMR CIRMF-SSM), Hôpital d´Instruction des Armées Omar BONGO ONDIMBA, Libreville, Gabon; 15 Centre Interdisciplinaire de Recherches Médicales de Franceville (CIRMF), Franceville, Gabon; 16 Molecular Bacteriology Laboratory, Institut Pasteur du Maroc, Casablanca, Morocco; 17 Department of Public Health and Clinical Research, Mohammed VI Center for Research and Innovation (CM6RI), Rabat, 10112 Morocco; 18 Mohammed VI International School of Public Health, Mohammed VI University of Sciences and Health, Casablanca, 82403 Morocco; 19 Higher Institute of Nursing Professions and Health Techniques, Ministry of Health and Social Protection, Rabat, Morocco; 20 Laboratory of Clinical Immunology, Infection and Autoimmunity (LICIA), Faculty of Medicine and Pharmacy, Hassan II University, Casablanca, 20250 Morocco; 21 Bacteriology-Virology and Hospital Hygiene Laboratory, Ibn Rochd University Hospital Centre, Casablanca, 20250 Morocco; 22 Faculty of Medicine and Dentistry, University of Sciences, Techniques and Technologies of Bamako (USTTB), Bamako, Mali; 23 Department of Pediatrics, Gabriel Touré University Hospital, Bamako, Mali; 24 Reproductive, Maternal, Newborn, and Child Health (ReMARCH) Research Unit, Research Institute of the Centre of Expertise and Biological Diagnostic of Cameroon (CEDBCAM-RI), Yaoundé, Cameroon; 25 Antimicrobial Research Unit, School of Health Sciences, College of Health Sciences, University of KwaZulu-Natal, Durban, South Africa; 26 Infection and Global Health Division, School of Medicine, University of St Andrews, St Andrews, UK; 27 Immunopathology-Immunotherapy-Immunomonitoring Laboratory, Mohammed VI Faculty of Medicine, Mohammed VI University of Health and Sciences, Casablanca, Morocco; 28 Anesthesia and Critical Care, Ibn Rochd University Hospital, Hassan II University of Casablanca, Casablanca, Morocco; 29 Immunology Laboratory, University Hospital Center Ibn Rochd, Casablanca, Morocco; 30 Kwame Nkrumah University of Science and Technology, Kumasi, Ghana; 31 Department of Pediatric Infectious Diseases and Clinical Immunology, Ibn Rushd University Hospital, Casablanca, Morocco; 32 Pediatric Department, Faculty of Medicine and Pharmacy of Marrakech, University Hospital Mohammed VI, Cadi Ayyad University, Marrakech, 40030 Morocco; 33 Internal Medicine Department, Mohammed VI University of Sciences and Health (UM6SS), Casablanca, Morocco; 34 Faculty of Pharmacy, University of Sciences, Techniques and Technologies of Bamako (USTTB), Bamako, Mali; 35 Mohammed VI Center for Research and Innovation (CM6RI), Rabat, 10112 Morocco

**Keywords:** Sepsis, Pediatric, Children under five, Africa

## Abstract

**Background:**

Sepsis remains a major global health threat, particularly among children under five years in low- and middle-income countries. Africa bears a disproportionate burden of sepsis-related morbidity and mortality. Comprehensive data on the etiology, prevalence, and mortality of pediatric sepsis in Africa remain limited. This systematic review and meta-analysis aimed to determine the causative pathogens, pooled prevalence, mortality, and risk factors of sepsis in African children under five years of age.

**Methods:**

Following PRISMA guidelines, we conducted a systematic search of PubMed, Web of Science, and Scopus for studies published from January 2000 to December 2024. Studies reporting microbiologically confirmed sepsis in children aged 0 to 60 months in Africa were included. Data were managed with Epi Info (version 7.2.7.0) and analyzed in R (version 4.4.2). Pooled prevalence and mortality were estimated using a random-effects model. Heterogeneity was assessed using *I*^2^ statistics, and publication bias was evaluated via funnel plots, Egger’s test, and the trim-and-fill method. Subgroup analyses were performed to identify sources of heterogeneity. The quality of original studies was assessed using the National Heart, Lung, and Blood Institute’s Quality Assessment Tools. This review was registered in PROSPERO (CRD42024621969).

**Results:**

Forty-six studies from 19 African countries, including 98,651 children and 59,521 pathogens, were analyzed. The most common pathogens were *Klebsiella pneumoniae* (25%), *Acinetobacter baumannii* (14.3%), and *Staphylococcus aureus* (13.2%). Gram-negative bacteria predominated (58.1%), with regional variations. The pooled prevalence of sepsis was 40.2% (95% CI: 26.8–55.4%), decreasing to 27.1% (95% CI: 15.5–43.0%) after adjustment for publication bias. Pooled mortality among clinically suspected cases was 16.3% (95% CI: 10.6–24.4%). Significant risk factors included prolonged rupture of membranes (OR 2.28; 95% CI: 1.30–4.00, *p* < 0.001), low birth weight (OR 2.16; 95% CI: 1.21–3.87, *p* < 0.001), and prematurity (OR 3.13; 95% CI: 1.94–5.08, *p* < 0.001).

**Conclusion:**

Sepsis in African children under five is highly prevalent and associated with substantial mortality, driven primarily by Gram-negative bacteria. Regional differences in pathogen distribution highlight the need for tailored antimicrobial strategies. These findings underscore the urgency of improving neonatal and pediatric care, enhancing diagnostic capacity, and implementing targeted prevention and management interventions to reduce sepsis burden in Africa.

**Clinical trial number:**

Not applicable.

**Supplementary Information:**

The online version contains supplementary material available at 10.1186/s12879-026-13350-2.

## Background

Sepsis remains a critical global health threat, characterized by high incidence and mortality rates. Annually, approximately 49 million cases are recorded, resulting in over 11 million sepsis-related deaths and accounting for nearly 20% of total global mortality [1]. The 2017 Global Burden of Sepsis study underscored stark disparities across demographic groups and geographic regions, revealing that nearly 85% of cases and deaths occur in low-to-middle sociodemographic settings, with sub-Saharan Africa bearing the most significant burden. Notably, pediatric populations are the most vulnerable, with children under five years of age accounting for 41.5% of all incident cases and 26.4% of sepsis-related fatalities globally [1].

The incidence of Sepsis follows a biphasic distribution, peaking significantly in early childhood and again in older age. Within the pediatric spectrum, neonatal sepsis, a systemic infection caused by bacterial, viral, or fungal pathogens in the first 28 days of life [2], remains a critical challenge for modern medicine. Together with preterm birth, it accounts for approximately 50% of all deaths among children under the age of five worldwide [3]. Clinical classification typically distinguishes between early-onset sepsis (EOS), occurring within the first 72 hours of life, and late-onset sepsis (LOS), manifesting thereafter [4, 5]. Very late-onset neonatal sepsis occurs in infants hospitalized beyond 30 days of life until discharge. While EOS is primarily associated with intrapartum vertical transmission frequently involving Group B *Streptococcus* and *Escherichia coli* [6, 7], LOS and very late-onset cases often stem from hospital-acquired or community-acquired infections. Although etiological profiles vary globally, Gram-negative organisms are increasingly identified as the predominant drivers of LOS in developing countries [6].

Despite the severe burden of sepsis in African children under five, comprehensive data regarding etiological pathogens and their regional distribution remain sparse. Evidence suggests that the etiology of sepsis is highly context-dependent, influenced by healthcare infrastructure, professional expertise, and resource availability. To address these critical knowledge gaps, this study aims to investigate the variations in etiology, prevalence, and clinical outcomes of pediatric sepsis across diverse African regions.

### Review questions

- What are the main causative pathogens of sepsis in children under five in Africa, and how are they distributed? What is the pooled prevalence of these pathogens?
- What is the prevalence of sepsis among children under five years old in Africa?
- What is the mortality rate of sepsis among children under five in Africa, and what are the associated risk factors?

## Methods

### Registration and protocol

This systematic review and meta-analysis was conducted in compliance with the Preferred Reporting Items for Systematic Reviews and Meta-Analyses (PRISMA) statement [8]. The study protocol, developed in accordance with PRISMA-P (Protocols) guidelines [9], was prospectively registered in the PROSPERO international database (CRD42024621969).

### Inclusion and exclusion criteria

The eligibility of studies was determined based on several predefined criteria. Regarding temporal and linguistic scope, inclusion was restricted to articles published in English or French from January 1, 2000, to December 31, 2024. This timeframe was selected to ensure clinical relevance, aligning with recent advances in understanding of pediatric sepsis and the expansion of diagnostic infrastructure in Africa since 2000, following the Millennium Development Goals. Studies were considered eligible if they reported on the causative pathogens of sepsis in pediatric populations, specifically children from birth to 60 months of age, within African countries.

A critical requirement for inclusion was microbiological confirmation; consequently, studies relying solely on clinical suspicion were excluded. The review accepted diverse diagnostic frameworks, including Sepsis-1 [10], Sepsis-2 [11], Sepsis-3 [12], the Phoenix pediatric for sepsis criteria [13], or other relevant definitions or criteria. Regarding study design, a wide range of quantitative methods was included, such as observational, cross-sectional, and longitudinal (prospective and retrospective cohort) studies, as well as randomized controlled trials. Conversely, qualitative research, case reports, reviews, editorials, commentaries, and clinical guidelines were excluded from the final analysis to ensure the robustness of the synthesized data.

### Information sources and search strategy

Two researchers (CHD and NDN) independently interrogated three primary electronic databases: PubMed, Scopus, and the Web of Science Core Collection. To maximize retrieval sensitivity, a structured search string was implemented across key domains, employing Boolean operators to refine the results. Specifically, “OR” was used to incorporate synonyms and related terms; “AND” was used to intersect distinct conceptual pillars, such as sepsis and African geographic regions; and “NOT” was used to exclude irrelevant literature. The Medical Subject Headings (MESH) terms and keywords used in the search were as follows: (Sepsis OR “septicem* OR “septicaem*” OR “Septic Shock) AND (Etiolog* OR aetiolog* OR Prevalence OR Outcomes) AND (Angola OR Algeria OR Benin OR Botswana OR “Burkina Faso” OR Burundi OR Cameroon OR “Cape Verde” OR “Central African Republic” OR Chad OR Comoros OR “Republic of the Congo” OR “Congo Brazzaville” OR “Democratic republic of the Congo” OR “Cote d’Ivoire” OR Djibouti OR “Equatorial Guinea” OR Egypt OR Eritrea OR Ethiopia OR Gabon OR “The Gambia” OR Ghana OR Guinea OR Guinea-Bissau OR Kenya OR Libya OR Lesotho OR Liberia OR Madagascar OR Malawi OR Mali OR Mauritania OR Mauritius OR Morocco OR Mozambique OR Namibia OR Niger OR Nigeria OR Reunion OR Rwanda OR “Sao Tome and Principe” OR Senegal OR Seychelles OR “Sierra Leone” OR Somalia OR “South Africa” OR Sudan OR Swaziland OR Eswatini OR Tanzania OR Togo OR Tunisia OR Uganda OR “Western Sahara” OR Zambia OR Zimbabwe). For the Web of Science Core Collection, age-specific keywords—including “Newborn”, “Neonate”, “Infant”, and “Preschool Child”—were integrated into the search string. In contrast, for PubMed and Scopus, database filters were used directly to restrict results to children under five years old and human subjects. These filters were further refined to align with the review’s objective by isolating relevant study designs. Finally, a manual “snowballing” search was conducted by screening the reference lists of all included articles to capture any additional eligible studies. The comprehensive search architecture for each database is provided in Supplementary Material 1. These databases were strategically selected for their rigorous indexing and comprehensive coverage of international and regional biomedical literature. This combination ensures highly reproducible searches, further supplemented by manual screening of the reference lists of all eligible studies.

### Study screening and selection

The screening process was conducted in two parallel, independent phases by two distinct reviewer groups (five members per group). Initially, duplicates were removed, and the lead coordinator of each group performed a preliminary screening of titles and abstracts against the predefined inclusion criteria. Following this initial phase, coordinators convened with their respective group members to consolidate findings and identify articles for full-text review.

**Fig. 1:**
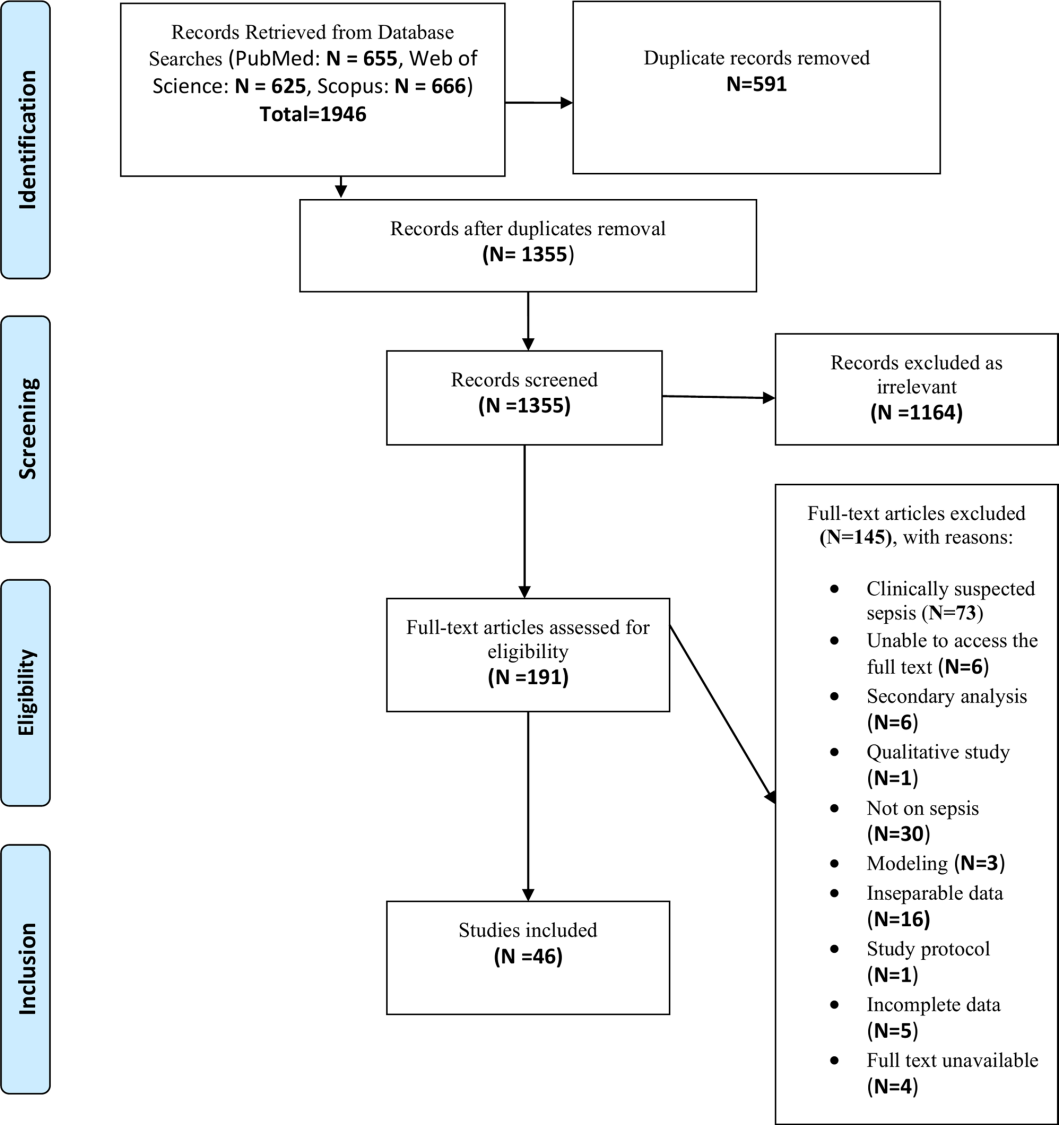
Flow diagram of study selection process

The eligibility of each potential study was then meticulously assessed through a comprehensive full-text analysis. To ensure the reliability of the final selection, the results from both reviewer groups were cross-referenced and reconciled. Any discrepancies or conflicting interpretations were resolved through internal consensus and discussion. The specific rationales for excluding studies during the full-text screening stage are detailed in Fig. 1

### Data extraction

Data extraction was performed by two investigators (CHD and IG) using standardized collection forms. Primary study characteristics and clinical variables were captured using Epi Info (version 7.2.7.0), while a dedicated Microsoft Excel (2021) spreadsheet was utilized to meticulously catalog causative pathogens and their respective frequencies. The extracted data encompassed a comprehensive range of variables, including: first author, study period, publication year, journal of publication, country and region of study, study setting, sample size, number of clinically suspected sepsis cases, number of culture proven sepsis cases, number of patients per age group, number of deaths, risk factors, infection site, causative pathogens and their frequencies.

### Risk of bias (quality) assessment

The methodological quality of the included studies was independently evaluated by two investigators (CHD and ID) using standardized criteria from the National Heart, Lung, and Blood Institute (NHLBI). Specifically, the Quality Assessment Tool for Observational Cohort and Cross-Sectional Studies, along with the Tool for Controlled Intervention Studies, were employed to assess the risk of bias [14]. Each study was rigorously appraised across 14 distinct domains. Based on the aggregate scores, studies were categorized into three quality tiers: **“Good”** for scores ≥ 11/14, **“Fair”** for scores between 8/14 and 10/14, and **“Poor”** for those scoring < 8/14. Any disagreements between the evaluators were resolved through discussion until a consensus was reached, ensuring the robustness of the quality ratings.

### Statistical analysis

Statistical analyses were performed using R (version 4.4.2), primarily utilizing the meta and metafor packages. The pooled prevalence of sepsis and its associated mortality rates were estimated using a random-effects model, specified a priori to account for anticipated clinical and methodological heterogeneity across the included studies. The results are presented via forest plots, displaying individual and pooled outcomes with their corresponding 95% confidence intervals (CIs). For etiological data, the pooled prevalence of specific pathogens was calculated as the proportion of each isolated pathogen relative to the total number of isolates.

Heterogeneity was assessed using the chi-square test and the *I*^*2*^ statistic, with a value of *I*^2^ > 75% or a significant Cochran’s Q test (*p* < 0.05) indicating substantial heterogeneity. To investigate the sources of this variance, univariable meta-regressions were conducted within a mixed-effects framework, along with subgroup analyses. These analyses examined several moderators, including country, geographic region, study design, sample size, clinical diagnostic criteria, and publication year, and assessed performance using regression coefficients, *p*-values, and the proportion of explained heterogeneity (*R*^*2*^).

Risk factors associated with sepsis were synthesized using odds ratios (ORs) and their 95% CIs. Potential publication bias was evaluated through funnel plots and Egger’s test [15]. To ensure the stability of our findings, a sensitivity analysis using the trim-and-fill method was performed, allowing estimation and adjustment of potential bias by imputing missing studies due to plot asymmetry [16].

## Results

### Search results and records selection

The initial systematic search across the three electronic databases yielded a total of 1,946 records, distributed as follows: 655 from PubMed, 625 from the Web of Science core collection, and 666 from Scopus. Following the removal of 591 duplicates, 1,355 unique citations underwent a preliminary screening of titles and abstracts. This phase resulted in the exclusion of 1,164 records that did not align with the predefined criteria. The remaining 191 articles underwent a full-text eligibility assessment. After a detailed review, 145 studies were excluded, leaving 46 for synthesis. The complete study selection process and the reasons for exclusion are detailed in Fig. 1.

### Study characteristics

A total of 46 studies were included, comprising 10 cross-sectional, 8 prospective cross-sectional, 8 retrospective, 8 prospective, 3 retrospective cross-sectional, 2 cohort, 2 prospective cohort, 1 randomized controlled trial, and 1 prospective observational study. These were conducted across 19 African countries. Geographically, 20 studies were conducted in Eastern Africa, 14 in Western Africa, 5 in Southern Africa, 4 in Northern Africa, and 3 in Central Africa. Notable representation was observed from Ethiopia (*n* = 11), Nigeria (*n* = 8), South Africa (*n* = 4), Uganda (*n* = 3), Egypt (*n* = 3), and Ghana (*n* = 3) (Table 1).

**Table 1:** Characteristics of the included studies

Author (Year) (ref)	Country	Region	Design	Sepsis diagnostic criteria	Sample size	Total pathogens	Percentage Confirmed pathogens (%)
Mwila Kabwe et al. (2016) [17]	Zambia	Eastern Africa	Cross sectional	EC	313	113	36.10
Dharshni Pillay et al. (2021) [18]	South Africa	Southern Africa	Retrospective	Other	681	681	100.00
Usmael Jibro et al. (2024) [19]	Ethiopia	Eastern Africa	Prospective Cohort	Other	415	21	5.06
Minichil Worku et al. (2022) [20]	Ethiopia	Eastern Africa	Cross sectional	EC	250	125	50.00
Endale Worku et al. (2022) [21]	Ethiopia	Eastern Africa	Prospective Cross-sectional	EC	392	143	36.48
Rudzani C Mashau et al. (2022) [22]	South Africa	Southern Africa	Retrospective Cross-sectional	EC	43438	43438	100.00
G Ahmed et al. (2023) [23]	Nigeria	Western Africa	Prospective observational	EC	167	16	9.58
Gashaw Amsalu et al. (2024) [24]	Ethiopia	Eastern Africa	Prospective Cross-sectional	IPSC	370	80	21.62
Abel Abera Negash et al. (2021) [25]	Ethiopia	Eastern Africa	Prospective Cross-sectional	IPSC	101	34	33.66
Sem Ezinmegnon et al. (2022) [26]	Benin	Western Africa	Prospective	EC	581	107	18.42
Anna Roca et al. (2023) [27]	Gambia/Burkina Faso	Western Africa	Randomized Controlled Trial	EC	157	88	56.05
Reenu Thomas et. (2024) [28]	South Africa	Southern Africa	Retrospective	Other	39391	10461	26.56
N S Mangeni et al. (2021) [29]	South Africa	Southern Africa	Retrospective	EC	1025	526	51.32
Guy M Mulinganya et al. (2021) [30]	Congo	Central Africa	Cross sectional	EC	150	50	33.33
Francis K Wuni et al. (2023) [31]	Ghana	Western Africa	Retrospective Cross-sectional	EC	276	37	13.41
Jean Claude Niyoyita et al. (2024) [32]	Rwanda	Eastern Africa	Retrospective Cross-sectional	Other	422	21	4.98
Tinuade A Ogunlesi et al. (2010) [33]	Nigeria	Western Africa	Retrospective	EC	527	174	33.02
Melkamu Berhane et al. (2021) [34]	Ethiopia	Eastern Africa	Cohort	WHO_PSBI	304	95	31.25
Gabriel Kambale Bunduki et al. (2019) [35]	Congo	Central Africa	Prospective Cross-sectional	IPSC	228	69	30.26
Andrea Nebbioso et al. (2021) [36]	Central African Republic	Central Africa	Prospective Cohort	EC	124	33	26.61
Henry Zamarano et al. (2021) [37]	Uganda	Eastern Africa	Cross sectional	Other	122	72	59.02
Demissie Shitaye et al. (2010) [38]	Ethiopia	Eastern Africa	-	EC	302	135	44.70
Abebe Sorsa (2019) [39]	Ethiopia	Eastern Africa	Prospective Cross-sectional	EC	303	79	26.07
Ruchika Kohli-Kochhar et al. (2011) [40]	Kenya	Eastern Africa	Retrospective	Other	665	156	23.46
Mulat Dagnew et al. (2013) [41]	Ethiopia	Eastern Africa	Retrospective Cross-sectional	Other	64	21	32.81
Ayoola et al. (2013) [42]	Nigeria	Western Africa	Prospective	EC	102	39	38.24
Kenneth C Iregbu et al. (2006) [43]	Nigeria	Western Africa	Retrospective	Other	390	85	21.79
Martin M Meremikwu et al. (2005) [44]	Nigeria	Western Africa	-	Other	1044	497	47.61
W.A. Seliem et al. (2018) [45]	Egypt	Africa Northern	Cross-sectional	EC	188	65	34.57
Fortress Yayra Aku et al. (2020) [46]	Ghana	Western Africa	Cross-sectional	Other	150	26	17.33
E.A. ADEJUYIGBE et al. (2001) [47]	Nigeria	Western Africa	Prospective	EC	119	69	57.98
Josephine Tumuhamye et al. (2020) [48]	Uganda	Eastern Africa	Cross-sectional	WHO_IMCI	359	46	12.81
Patricia Perez-Palacios et al. (2023) [49]	Morocco	Africa Northern	Retrospective	Other	524	199	37.98
Daniel Geleta et al. (2024) [50]	Ethiopia	Eastern Africa	Cohort	EC	342	138	40.35
Tsehaynesh G/Eyesus et al. (2017) [51]	Ethiopia	Eastern Africa	Prospective Cross-sectional	Other	251	120	47.81
Neema Kayange et al. (2010) [52]	Tanzania	Eastern Africa	Prospective Cross-sectional	WHO_PSBI	300	149	49.67
J Mugalu et al. (2006) [53]	Uganda	Eastern Africa	-	WHO_IMCI	293	110	37.54
Eman M Rabie Shehab El-Din et al. (2015) [54]	Egypt	Africa Northern	Prospective	EC	344	140	40.70
Pius Simon et al. (2016) [55]	Nigeria	Western Africa	Prospective	EC	110	46	41.82
Zoly Nantenaina Ranosiarisoa et al. (2019) [56]	Madagascar	Eastern Africa	Prospective	WHO_IMCI	307	132	43.00
Lydia Mudzikatib et al. (2015) [57]	Botswana	Southern Africa	Cross-sectional	Other	909	249	27.39
Francis Kwame Morgan Tetteh et al. (2022) [58]	Ghana	Western Africa	Cross-sectional	Other	471	153	32.48
Andreas Chiabi et al. (2011) [59]	Cameroon	Western Africa	Prospective	EC	218	42	19.27
J Seni et al. (2019) [60]	Tanzania	Eastern Africa	Cross-sectional	WHO_YISG	950	135	14.21
Lamiaa Mohsen et al. (2017) [4]	Egypt	Africa Northern	Prospective	EC	314	245	78.03
Olugbenga A Mokuolu et al., (2002) [61]	Nigeria	Western Africa	Retrospective	Other	198	61	30.81

The synthesized data encompassed 98,651 patients, from whom 59,521 pathogens were identified. All studies were published between 2001 and 2024 and were conducted in hospital settings, with only one study specifically addressing community-acquired sepsis [25]. The vast majority of studies focused exclusively on neonatal sepsis (birth to 1 month), whereas six studies reported data on post-neonatal sepsis in children up to five years of age.

### Diagnostic criteria and age distribution

Diagnostic criteria for sepsis varied widely across the 46 included studies. Only nine studies used international consensus definitions, such as the International Pediatric Sepsis Consensus (IPSC, *n* = 3) [62] or specific World Health Organization (WHO) guidelines. These include the Possible Serious Bacterial Infection in Young Infants guideline (WHO_PSBI, *n* = 2) [63], the Integrated Management of Childhood Illness guideline (WHO_IMCI, *n* = 3) [64], and the WHO Young Infants Study Group (YISG, *n* = 1) [65]. Conversely, 22 studies relied on empirical clinical (EC) definitions, using various combinations of signs such as fever or hypothermia, feeding difficulties, lethargy, respiratory distress, convulsions, and neurological or hemodynamic abnormalities (EC; empirical clinical). These signs overlap but differ in number and combination across studies. Lastly, 15 studies provided no detailed clinical criteria or reference to a consensus definition, instead reporting case identification based on clinician judgment or clinical suspicion without explicit standardization (Other) (Table 1). This lack of consensus highlights a broader gap in standardized diagnostic approaches.

The patient population was heavily skewed toward the neonatal period (birth to 1 month), comprising 97,240 patients (over 98% of the total sample). In contrast, infants (1 month to 1 year) and preschool-aged children (1 to 5 years) were underrepresented, with 643 and 768 patients, respectively. While this focus highlights the critical priority of neonatal sepsis, it also reveals a substantial knowledge gap regarding the post-neonatal period up to five years of age within the African context.

### Leading pathogens of sepsis

The etiology of sepsis was documented in all 46 studies, with a total of 59,521 pathogens identified. This included 54,998 bacteria (92.4%), 4,500 fungi (7.5%), and 23 viruses (0.04%) as detailed in Table 2.

**Table 2:** Leading pathogens of sepsis

Pathogens		isolate	Frequency	Percentage (%)		
				Sub-group*	Group**	Overall***
Bacteria (54998)	Gram negative (31956)	*Klebsiella pneumoniae*	13787	43.14	25	23.16
		*Acinetobacter baumannii*	7840	24.53	14.25	13.17
		*Escherichia coli*	3095	9.68	5.62	5.19
		*Enterobacter cloacae*	1609	5.03	2.92	2.70
		*Serratia marcescens*	1501	4.69	2.72	2.52
		*Pseudomonas aeruginosa*	974	3.04	1.77	1.63
		*Klebsiella species*	129	0.40	0.23	0.21
		*Acinetobacter species*	93	0.29	0.16	0.15
		*Enterobacter species*	62	0.19	0.11	0.10
		*Klebsiella oxytoca*	45	0.14	0.08	0.07
		*Pseudomonas species*	41	0.12	0.07	0.06
		*Proteus mirabilis*	31	0.09	0.05	0.05
		*Enterobacter hormaechei*	30	0.09	0.05	0.05
		*Klebsiella ozanae*	29	0.09	0.05	0.04
		*Salmonella species*	24	0.07	0.04	0.04
		*Chromobacterium species*	22	0.06	0.04	0.03
		*Citrobacter species*	20	0.06	0.03	0.03
		*Acinetobacter lwoffii*	15	0.04	0.02	0.02
		*Stenotrophomonas maltophilia*	9	0.02	0.01	0.01
		*Enterobacter aerogenes*	9	0.02	0.01	0.01
		*Burkholderia cepacia*	9	0.02	0.01	0.01
		*Serratia species*	8	0.02	0.01	0.01
	Gam positive (23042)	*Staphylococcus aureus*	7257	31.49	13.19	12.19
		*Coagulase negative staphylococci*	4267	18.51	7.75	7.16
		*Enterococcus faecium*	3655	15.86	6.64	6.14
		*Enterococcus faecalis*	3520	15.27	6.40	5.91
		Group B *Streptococcus*	2807	12.18	5.10	4.71
		*Streptococcus viridans*	307	1.33	0.55	0.51
		*Enterococcus species*	181	0.78	0.32	0.30
		*Streptococcus species*	102	0.44	0.18	0.17
		*Listeria monocytogenes*	42	0.18	0.07	0.07
		*Streptococcus pyogenes*	34	0.14	0.06	0.05
		*Bacillus species*	29	0.12	0.05	0.04
		*Streptococcus pneumoniae*	25	0.10	0.04	0.04
		*Micrococcus species*	18	0.07	0.03	0.03
		*Corynebacterium species*	17	0.07	0.03	0.02
Fungi (4500)		*Candida parapsilosis*	1786		39.68	3
		*Candida albicans*	1475		32.77	2.47
		*Candida glabrata*	126		2.80	0.21
		*Candida auris*	82		1.82	0.13
		*Candida species*	57		1.26	0.09
Viruses (18)		Rhinovirus/Enterovirus	12		52.17	0.000202
		Respiratory Syncytial Virus	7		30.43	0.000118
		Coronavirus OC43	2		8.69	3.36E-05
		Norovirus GI/GII	1		4.34	1.68E-05
		Rotavirus A	1		4.34	1.68E-05

Percentages are shown as follows: * within bacterial subgroups (Gram-negative or Gram-positive); ** within microbial groups (bacteria, fungi, or viruses); *** within all isolates (overall). Rare pathogens with very low isolate counts are not displayed in the main table but are included in the dataset. This includes Gram-negative pathogens with ≤7 isolates (*Enterobacter agglomerans (7 isolates)*, *Citrobacter freundii (6 isolates)*, *Haemophilus influenzae (4 isolates)*, *Providencia species (4 isolates)*, *Aeromonas species (3 isolates)*, *Moraxella catarrhalis (3 isolates)*, *Acrobacterium species (2 isolates)*, *Shigella species (2 isolates)*, *Ochrobactrum anthropi (2 isolates)*, *Kluyvera species (2 isolates)*, *Campylobacter species (2 isolates)*, *Raoultella species (2 isolates)*, *Pasteurella species (1 isolates)*, *Chryseomonas luteola (1 isolates)*, *Weeksella virosa (1 isolates)*, *Brevundimonas vesicularis (1 isolates)*, *Hafnia alvei (1 isolates)*, *Morganella morganii (1 isolates)*, *Clostridium species (1 isolates)*, *Citrobacter koseri (1 isolates)*, *Enterobacter kobei (1 isolates)*, *Citrobacter youngae (1 isolates)*, *Neisseria species (1 isolates)* and Gram-positive pathogens with ≤6 isolates (*Diphteroides (6 isolates)*, *Kocuria species (3 isolates)*, *Streptococcus mitis (2 isolates)*, *Streptococcus oralis (2 isolates)*, *Streptococcus anginosus (1 isolates)*, *Listeria species (1 isolates*). Additionally, Other Gram-negative pathogens (2,275 isolates), Coliforms (134 isolates), Other Gram-positive pathogens (782 isolates), and other yeasts (974) were identified only at the group level and are not shown in the main table

Among the bacterial isolates, Gram-negative bacteria were predominant, accounting for 58.1% (31,956/54,998), whereas Gram-positive bacteria accounted for 41.8% (23,042/54,998). The most frequently encountered bacterial pathogens were *Klebsiella pneumoniae* (*n* = 13,787; 25%), *Acinetobacter baumannii* (*n* = 7,840; 14.25%), *Staphylococcus aureus* (*n* = 7,257; 13.19%), and Coagulase-negative *staphylococci* (*n* = 4,267; 7.7%). Other notable isolates included *Enterococcus faecium* (*n* = 3,655; 6.6%), *Enterococcus faecalis* (*n* = 3,520; 6.4%), *Escherichia coli* (*n* = 3095; 5.6%), Group B *streptococcus* (*n* = 2807; 5.1%), *Enterobacter cloacae* (*n* = 1609; 2.9%), *Serratias Marcescens* (1501; 2.7%), and *Pseudomonas aeruginosa* (*n* = 974, 1.7%). Unidentified Gram-negative and Gram-positive pathogens were also reported, accounting for 2,275 (4.1%) and 782 (1.4%) isolates, respectively.

Regarding fungal isolates, *Candida* species and unidentified yeasts (*n* = 974) were reported. *Candida* species accounted for 78.8% of all fungal isolates (3,526/4,500). Within this genus, *Candida parapsilosis* was the most prevalent at 39.6% (*n* = 1,786), followed by *Candida albicans at* 32.7% (*n* = 1,475), *Candida glabrata at* 2.8% (*n* = 126), *Candida auris at* 0.6% (*n* = 28), and other unspecified *Candida* species at 1.2% (*n* = 57).

Viral infections were reported in only one study [26], which identified 23 viruses. These included Rhinovirus/Enterovirus (*n* = 12), Respiratory Syncytial Virus (*n* = 7), Coronavirus OC43 (*n* = 2), Rotavirus A (*n* = 1), and Norovirus GI/GII (*n* = 1).

### Subgroup analysis of leading pathogens

**Fig. 2:**
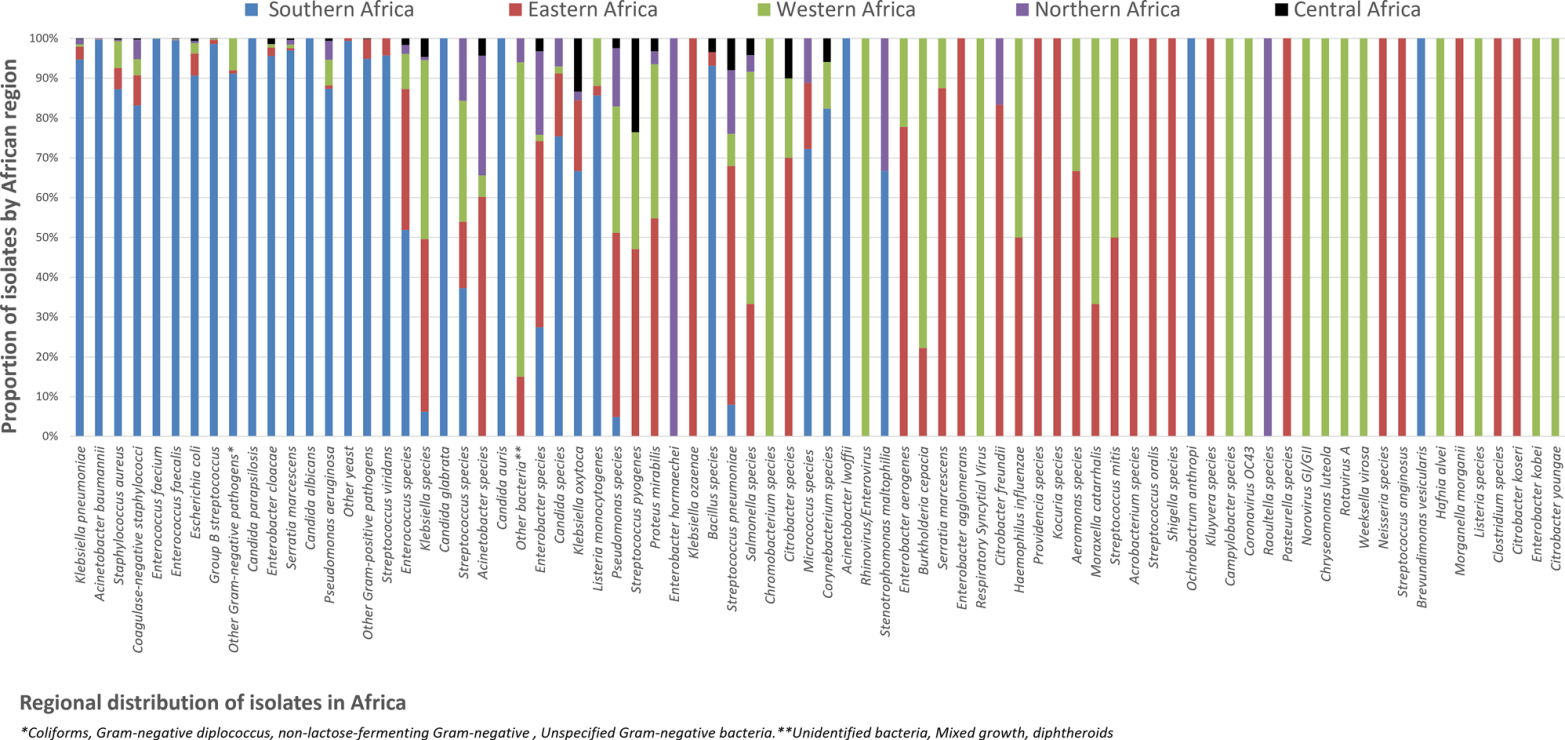
Regional distribution of clinical isolates in Africa.*coliforms, Gram-negative diplococcus, non-lactose-fermenting Gram-negative bacteria, unspecified Gram-negative pathogens. **unidentified bacteria, mixed growth, diphtheroids

The distribution of pathogens exhibited regional variation across the continent, as illustrated in Fig. 2. In nearly all African regions, Gram-negative bacteria were more frequently reported than Gram-positive bacteria. The notable exception was Western Africa, where Gram-positive bacteria were predominant.

### Pathogen distribution in Central Africa

**Fig. 3:**
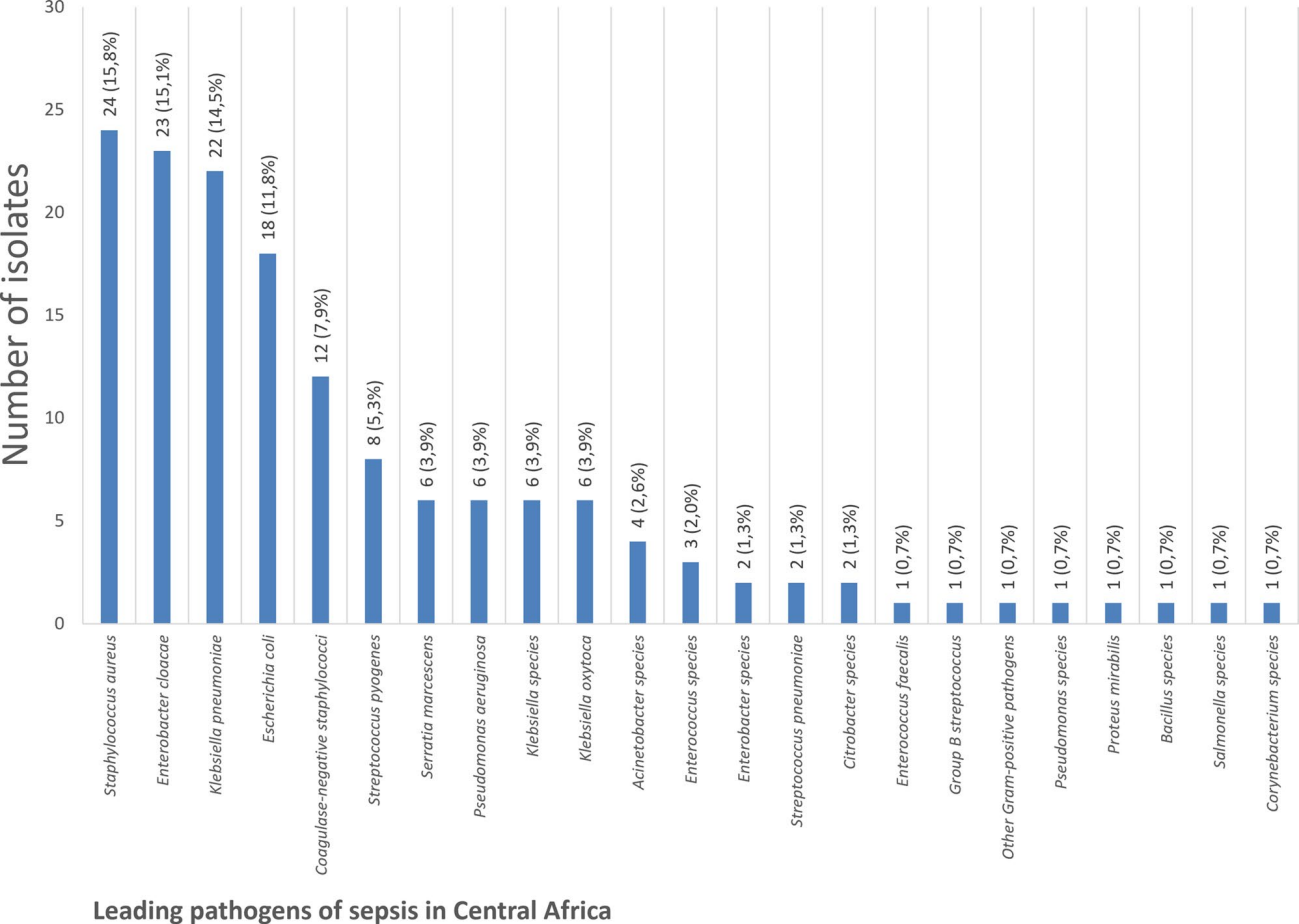
Leading pathogens of sepsis in Central Africa

A total of 152 bacterial species were identified across the Central African region. Gram-negative bacteria constituted the majority, accounting for 98 isolates (64.5%), whereas Gram-positive species accounted for 54 isolates (35.5%). The prevalent pathogens were *Staphylococcus aureus* (*n* = 24), followed by *Enterobacter cloacae* (*n* = 23), *Klebsiella pneumoniae* (*n* = 22), and *Escherichia coli* (*n* = 18). Other identified organisms included coagulase-negative *staphylococci* (*n* = 12) and *Streptococcus pyogenes* (*n* = 8). Furthermore, *Serratia marcescens*, *Pseudomonas aeruginosa*, and *Klebsiella oxytoca* each accounted for six isolates, as did unspecified *Klebsiella species*. Lower frequencies were observed for other bacteria, such as *Acinetobacter species, Enterococcus species*, *Enterobacter species*, *Citrobacter species*, and *Streptococcus pneumoniae*. The regional microbial landscape is illustrated in Fig. 3.

### Pathogen distribution in Eastern Africa

**Fig. 4:**
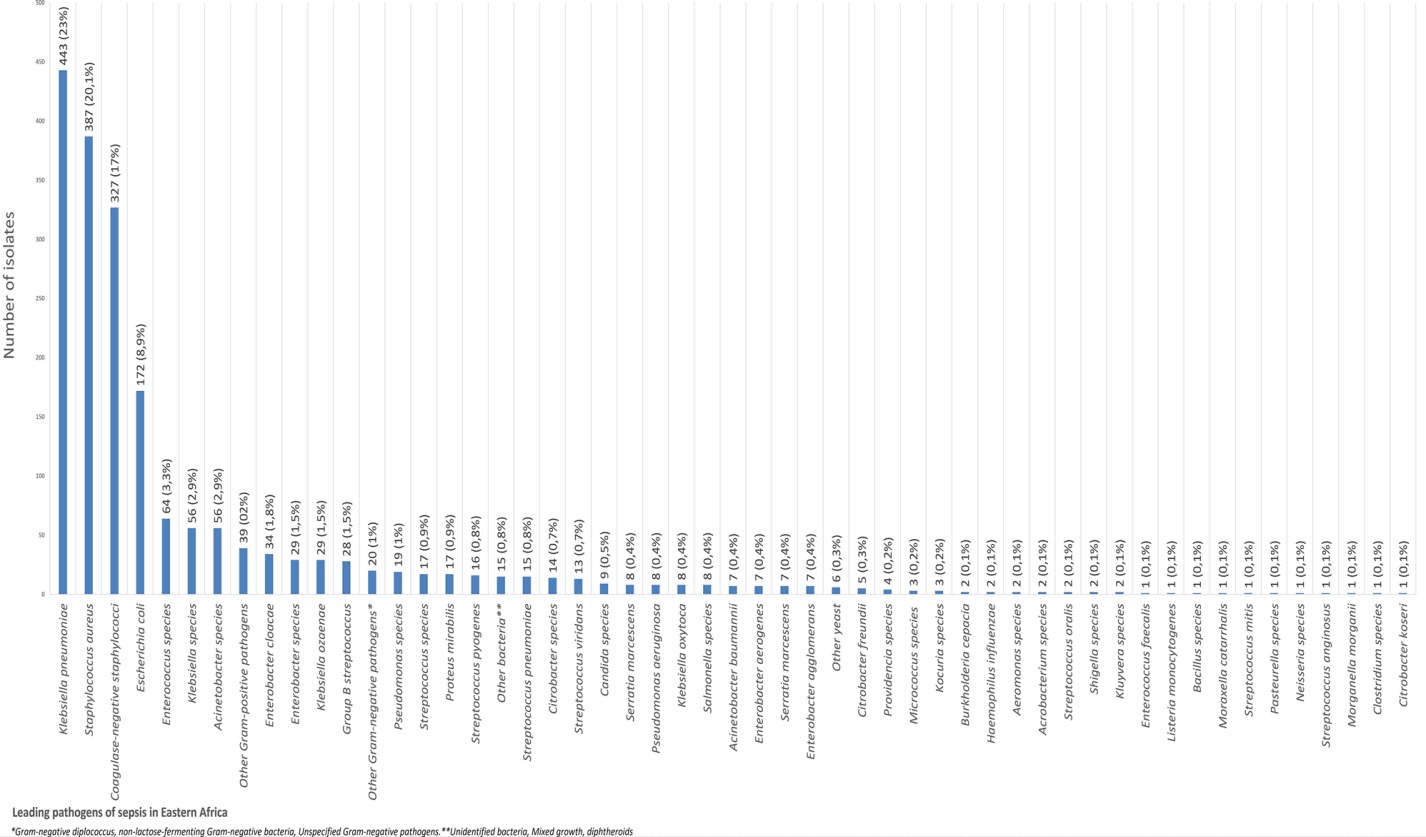
Leading pathogens of sepsis in Eastern Africa. *Gram-negative diplococcus (*n* = 1), non-lactose-fermenting Gram-negative bacteria (*n* = 4), unspecified Gram-negative pathogens (*n* = 15).**Unidentified bacteria (*n* = 6), mixed growth (*n* = 3), diphtheroids (*n* = 6)

In Eastern Africa, a total of 1,910 bacterial isolates were documented, showing a nearly balanced distribution between Gram-negative bacteria (*n* = 989; 51.7%) and Gram-positive bacteria (*n* = 921; 48.2%) (Fig. 4). The primary bacterial species identified bacterial species were, *Klebsiella pneumoniae* (*n* = 443), *Staphylococcus aureus* (*n* = 387), and Coagulase-negative *Staphylococci* (*n* = 327) followed by *Escherichia coli* (*n* = 172). Moderate frequencies were reported for *Enterococcus* species (*n* = 64), while *Klebsiella* and *Acinetobacter* species accounted for 56 isolates each.

Less frequently reported organisms included Group B *Streptococcus*, *Pseudomonas* species, *Proteus mirabilis*, and *Streptococcus pyogenes*. In addition to the bacterial burden, 15 fungal isolates were identified, consisting primarily of *Candida* species (*n* = 9) and other yeasts (*n* = 6). The comprehensive microbial profile for this region is summarized in Fig. 4.

### Pathogen distribution in Northern Africa

In Northern Africa, a total of 645 bacterial isolates were documented, comprising 371 (57.51%) Gram-negative bacteria and 274 (42.4%) Gram-positive bacteria. The microbial landscape was characterized by a high prevalence of *Coagulase-negative staphylococci* (*n* = 209) and *Klebsiella pneumoniae* (*n* = 181). These were followed by *Pseudomonas aeruginosa* (*n* = 46), *Enterobacter hormaechei* (*n* = 30), and *Staphylococcus aureus* (*n* = 29).

**Fig. 5:**
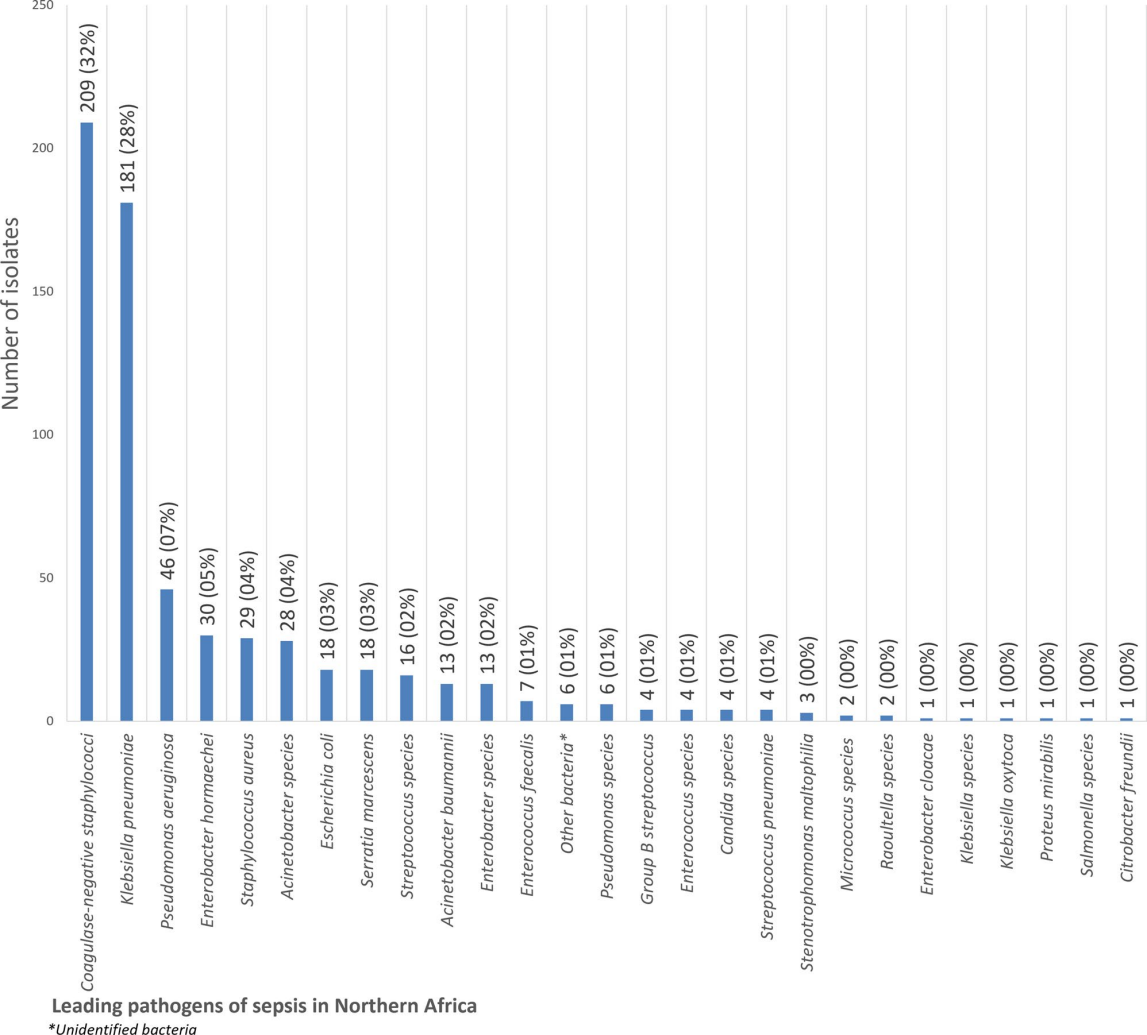
Leading pathogens of sepsis in Northern Africa. **unidentified bacteria (n = 6)

Other notable isolates included *Acinetobacter species* (*n* = 28), while *Escherichia coli* and *Serratia marcescens* accounted for 18 isolates each. Pathogens reported with lower frequency included *Enterococcus faecalis*, *Pseudomonas* species, *Group B Streptococcus*, *Streptococcus pneumoniae*, and *Stenotrophomonas maltophilia*. Additionally, fungal involvement was minimal, with only four isolates of *Candida species*. The detailed distribution of these pathogens is presented in Fig. 5.

### Pathogen distribution in Western Africa

In Western Africa, a total of 1,416 bacterial isolates were documented. Notably, this region was unique in its microbial profile, being the only geographic area in which Gram-positive bacteria predominated, accounting for 758 isolates (53.5%) compared with 658 Gram-negative isolates (46.4%).

**Fig. 6:**
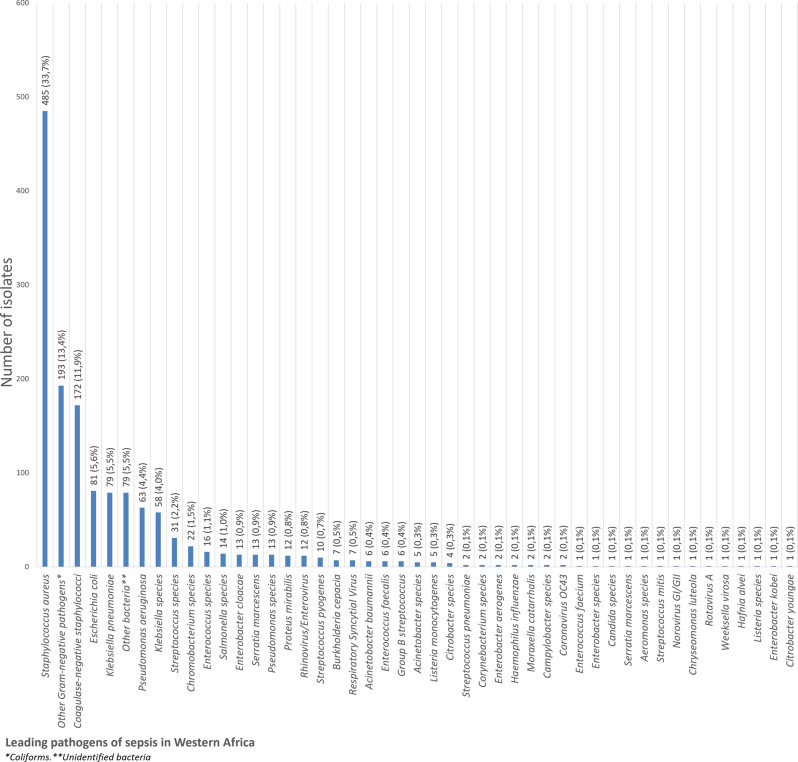
Leading pathogens of sepsis in Western Africa. *coliforms (*n* = 134). **Unidentified bacteria (*n* = 79)

The most frequently identified species was *Staphylococcus aureus* (*n* = 485), followed by Coagulase-negative *staphylococci* (*n* = 172). Among the Gram-negative isolates, Coliforms (*n* = 134), *Escherichia coli* (*n* = 81), and *Klebsiella pneumoniae* (*n* = 79) were the most prevalent. This distinct regional pattern of bacterial predominance is illustrated in Fig. 6.

### Pathogen distribution in Southern Africa

Southern Africa provided the most extensive dataset, comprising 50,875 bacterial isolates, with Gram-negative bacteria predominating (58.6%, *n* = 29,840) over Gram-positive species (41.3%, *n* = 21,035). The bacterial landscape was dominated by *Klebsiella nthropic* (*n* = 13,062), *Acinetobacter baumannii* (*n* = 7,814), and *Staphylococcus aureus* (*n* = 6,332). Other high-prevalent pathogens included *Enterococcus faecium* (*n* = 3,654), followed by *Coagulase-negative* staphylococci (*n* = 3,547), *Enterococcus faecalis* (*n* = 3,505), *Escherichia coli* (*n* = 2,806), and *Group B Streptococcus* (*n* = 2,768).

Minor bacterial isolates included *Enterobacter cloacae*, *Serratia marcescens*, *Pseudomonas aeruginosa*, and *Streptococcus viridans*, with rare occurrences of *Listeria monocytogenes*, *Acinetobacter lwoffii*, and *Ochrobactrum nthropic.*

**Fig. 7:**
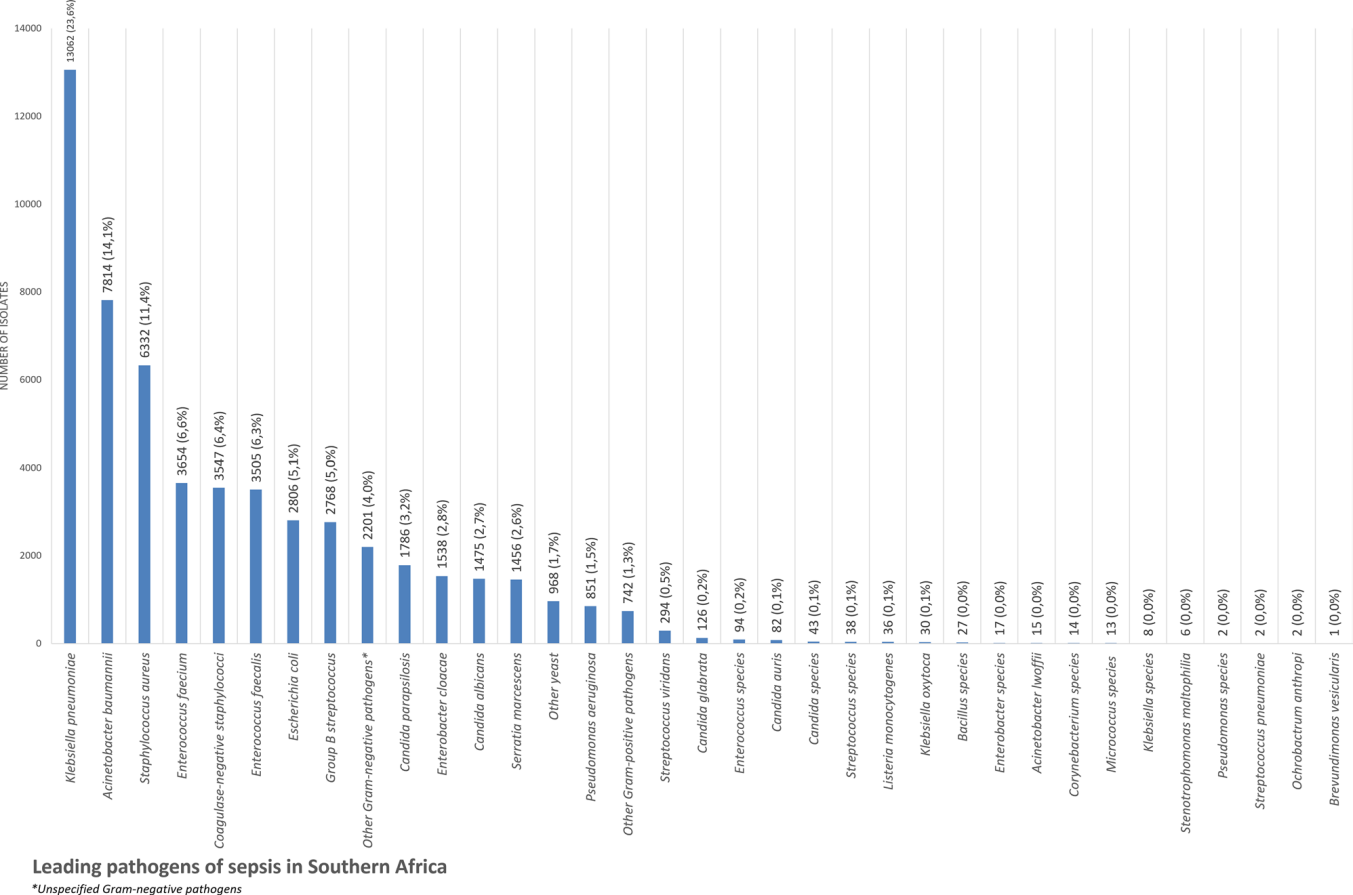
Leading pathogens of sepsis in Southern Africa. *unspecified Gram-negative pathogens (*n* = 2201)

Furthermore, the region reported a substantial fungal burden (*n* = 4,480), primarily driven by *Candida parapsilosis* (*n* = 1,786) and *Candida albicans* (*n* = 1,475), followed by unidentified yeasts and other *Candida* species, including *C. glabrata* and *C. auris*. The comprehensive microbial profile for this region is summarized in Fig. 7.

### Prevalence of sepsis

**Fig. 8:**
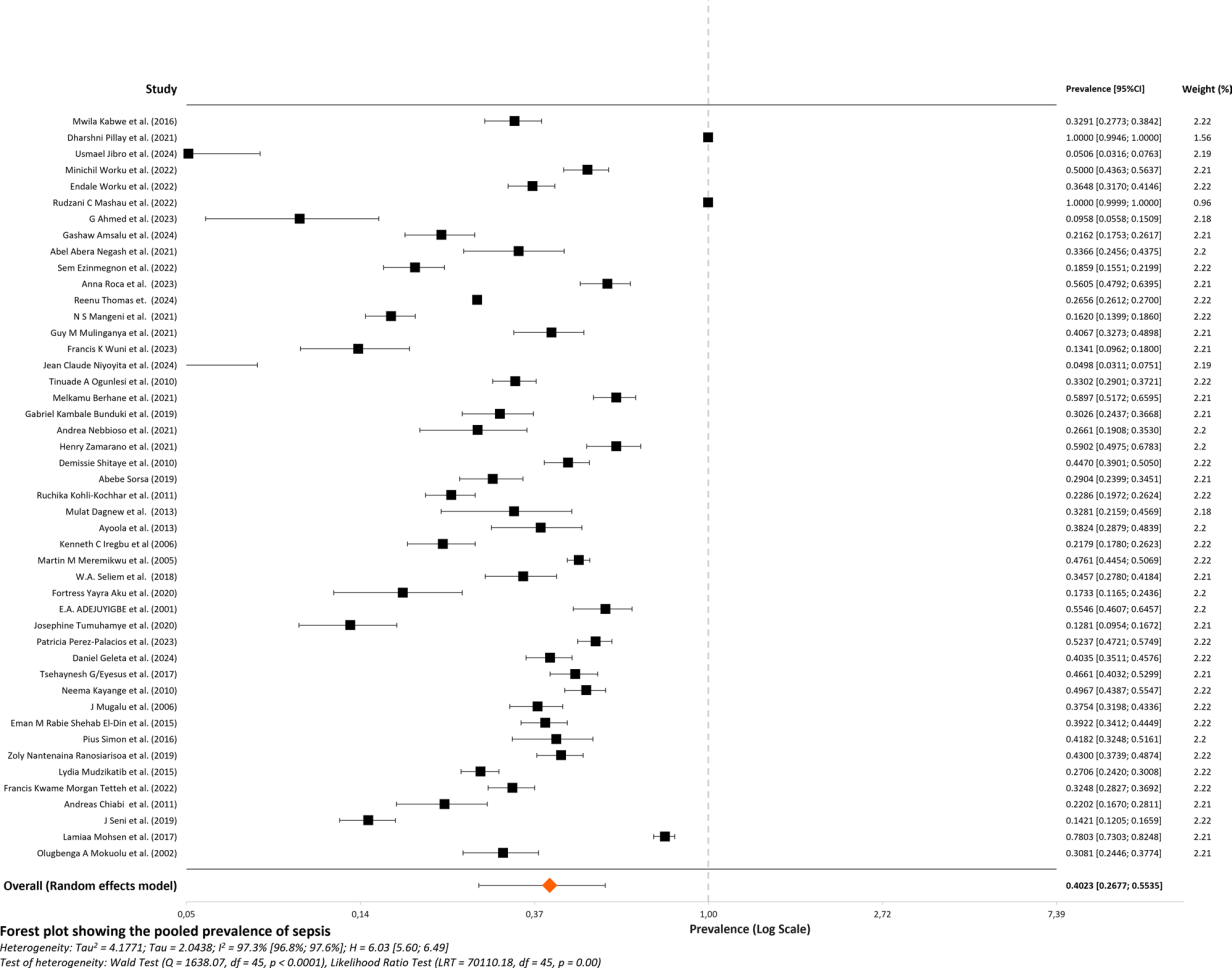
Forest plot showing the pooled prevalence of sepsis in Africa. Heterogeneity: Tau^2^ = 4.1771; tau = 2.0438; I^2^ = 97.3% [96.8%; 97.6%]; H = 6.03 [5.60; 6.49]. Test of heterogeneity: Wald test (Q = 1638.07, df = 45, *p* < 0.0001), likelihood ratio test (LRT = 70110.18, df = 45, *p* = 0)

All 46 studies included in this systematic review contributed data for the prevalence analysis. The overall pooled prevalence of sepsis was estimated at 40.23% (95% CI: 26.8% to 55.4%) as illustrated in Fig. 8.

A very high level of heterogeneity was observed across studies (*I*^*2*^ = 97.3%), a finding confirmed by a significant Cochran’s Q test (*p* < 0.001). This substantial variability indicates significant fluctuations in prevalence estimates, likely attributable to disparities in study settings, designs, patient populations, and diagnostic methodologies. Such considerable heterogeneity justified the a priori selection of a random-effects model to provide a more conservative and representative pooled estimate.

### Reporting biases

**Fig. 9:**
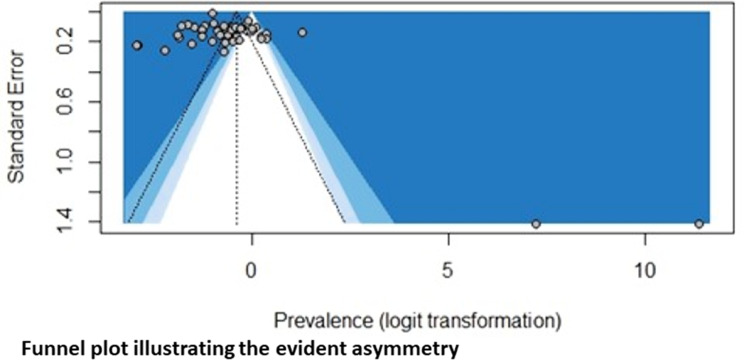
Funnel plot illustrating the evident asymmetry

Potential publication bias was assessed across all 46 included studies. Egger’s test indicated significant asymmetry (*t* = 2.33, *df* = 44, *p* = 0.024), with an intercept estimate of 2.57 (*SE* = 1.10). This statistical evidence of asymmetry, visualized in Fig. 9, suggests the presence of publication bias or other systematic influences, such as small-study effects or methodological heterogeneity.

The observed bias potential compromises the robustness of the raw pooled estimate. Consequently, to account for these missing studies and enhance the reliability of the findings, the ‘Trim-and-Fill’ procedure was applied as an adjustment in the meta-analytic method.

### Adjusted meta-analytic methods trim-and-fill procedure

To account for the potential publication bias indicated by the funnel plot asymmetry, the trim-and-fill method was applied to the 46 studies (Supplementary Material 2). This procedure imputed 9 additional studies to achieve symmetry. After this adjustment, the pooled prevalence of sepsis decreased from 40.23% to 27.1% (95% CI: 15.5% to 43.0%). This substantial reduction highlights a potential overestimation of the original effect size due to missing, small, or negative studies. However, residual heterogeneity remained very high (*I*^2^ = 97.9%), indicating that substantial variability remains unexplained by publication bias alone.

### Subgroup analysis

To investigate the observed clinical and methodological heterogeneity, comprehensive subgroup analyses were performed. The distribution of studies and detailed prevalence estimates across each subgroup is summarized in Table 3.

**Table 3:** Subgroup analysis of sepsis prevalence showing pooled estimates, confidence intervals, and heterogeneity metrics

Moderators	Subgroup	No. of studies	Prevalence (CI: 95%)	*I*^2^, *p*-value
Regions	Eastern Africa	20	33.6 (26.4–40.7)	70.49, <0.001
	Southern Africa	5	54.0 (16.9–91.0)	98.24, 0.004
	Western Africa	14	31.0 (23.2–38.9)	98.01, <0.001
	Central Africa	3	32.4 (24.5–40.3)	100.00, <0.001
	Northern Africa	4	51.1 (32.0–70.3)	97.13, <0.001
Sample size	1–1000	42	35.5 (29.7–41,3)	99.2, <0.001
	>1000	4	47.6 (11.0–84,2)	99.99, 0.011
Study design	Cross sectional	10	31.8 (22.5–41.1)	97.7, <0.001
	Retrospective	8	38.0 (19.0–57.0)	99.97, <0.001
	Prospective Cohort	2	15.5 (−5.6–36.6)	96.03, 0.151
	Prospective Cross sectional	8	35.3 (27.8–42.8)	91.72, <0.001
	Retrospective Cross sectional	3	37.8 (−4.6–80.3)	99.92, 0.081
	Prospective observational	1	9.6 (4.8–14.3)	—
	Prospective	8	42.0 (28.8–55.2)	97.66, <0.001
	Randomized Controlled Trial	1	56.0 (48.0–64.1)	—
	Cohort	2	49.5 (31.3–67.7)	94.07, <0.001
	Not reported	3	43.6 (37.7–49.5)	78.10, <0.001
Country	Benin	1	18.6 (15.5–22.0)	—
	Botswana	1	27.1 (24.2–30.1)	—
	Cameroon	1	22.0 (16.7–28.1)	—
	Central African Republic	1	26.6 (19.1–35.3)	—
	Congo	2	35.1 (24.9–45.3)	75.0, 0.045
	Egypt	3	50.7 (23.6–77.7)	98.6, <0.001
	Ethiopia	11	36.2 (27.3–45.1)	96.9, <0.001
	Gambia/Burkina Faso	1	56.1 (47.9–64.0)	—
	Ghana	3	21.1 (9.6–32.7)	94.1, <0.001
	Kenya	1	22.9 (19.7–26.2)	—
	Madagascar	1	43.0 (37.4–48.7)	—
	Morocco	1	52.4 (47.2–57.5)	—
	Nigeria	8	34.5 (24.4–44.7)	96.6, <0.001
	Rwanda	1	5.0 (3.1–7.5)	—
	South Africa	4	60.7 (16.0–105.4)	100.0, <0.001
	Tanzania	2	31.8 (−2.9–66.6)	99.2, <0.001
	Uganda	3	36.2 (10.1–62.3)	98.4, <0.001
	Zambia	1	32.9 (27.7–38.4)	—

The prevalence of sepsis varied significantly across African regions. The highest prevalence was reported in Southern Africa, 54.0% (95% CI: 16.9% to 91.0%), followed closely by Northern Africa at 51.1% (95% CI: 32.0% to 70.3%). In contrast, prevalence estimates for Eastern, Western, and Central Africa were lower, ranging from 31% to 33.6%.

At the national level, the highest rates were observed in South Africa at 60.7% (95% CI: 16.0% to 105.4%), followed by Gambia/Burkina Faso at 56.1% (95% CI: 47.9% to 64.0%), and Morocco at 52.4% (95% CI: 47.2% to 57.5%), while Rwanda reported the lowest prevalence, at 5.0% (95% CI: 3.1% to 7.5%).

Regarding sample size, studies with fewer than 1,000 participants reported a prevalence of 35.5% (95% CI: 29.7% to 41.3%), whereas larger studies (>1,000 patients) showed a higher estimate of 47.6% (95% CI: 11.0% to 84.2%)

Regarding study design, randomized controlled trials (56.0%, 95% CI: 48.0% to 64.1%) and cohort studies (49.5%, 95% CI: 31.3% to 67.7%) yielded the highest prevalence estimates. Prospective studies reported a prevalence of 42.0% (95% CI: 28.8% to 55.2%) followed by retrospective (38.0%, 95% CI: 19.0% to 57.0%) and cross-sectional (31.8%, 95% CI: 22.5% to 41.1%) designs. Other methodologies, such as prospective observational studies, yielded significantly lower estimates (9.6%; 95% CI: 4.8% to 14.3%).

### Meta-regression analysis

To further investigate the sources of the substantial heterogeneity observed, a univariable meta-regression was conducted encompassing all 46 studies. We assessed several variables as potential sources of heterogeneity, including year of publication (*p* = 0.645; *R*^*2*^ = 0.00%), geographic region (*p* = 0.113; *R*^*2*^ = 7.56%), study country (*p* = 0.701; *R*^*2*^ = 0.00%), study design (*p* = 0.707; *R*^*2*^ = 0.00%), clinical diagnostic criteria (*p* = 0.626; *R*^*2*^ = 0.00%) and sample size (*p* = 0.041; *R*^*2*^ = 6.77%).

**Fig. 10:**
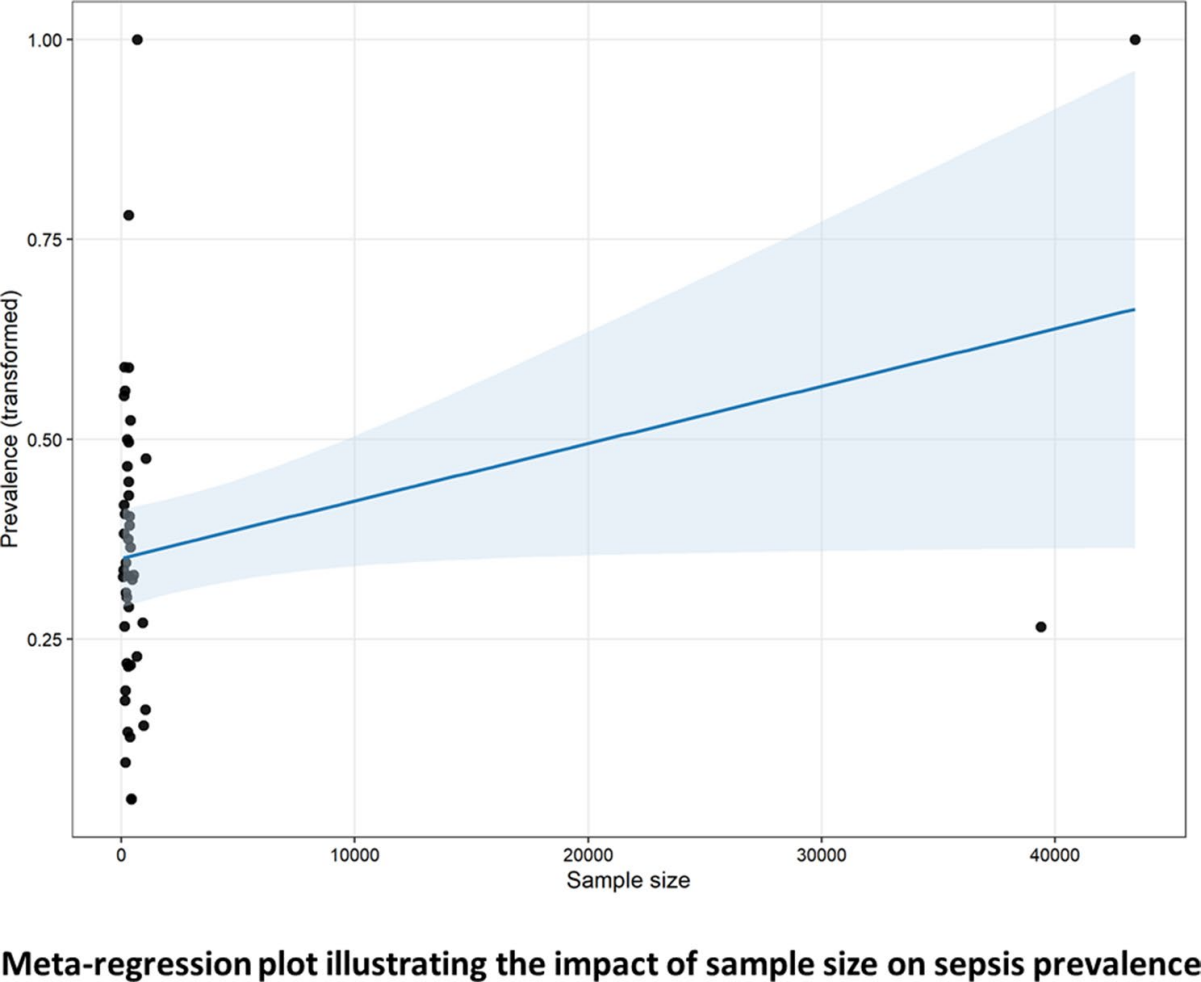
Meta-regression plot illustrating the impact of sample size on sepsis prevalence

Among these factors, only sample size was significantly associated with the observed heterogeneity (*p* < 0.05) as illustrated in Fig. 10. However, it explained a relatively small proportion of the total variance (*R*^*2*^ = 6.77%). Notably, the moderator Geographic region, although not statistically significant (*p* = 0.113), accounted for a slightly higher proportion of the variance (*R*^*2*^ = 7.56%), suggesting a potential regional influence on prevalence rates. The other variables did not significantly contribute to the heterogeneity. The remaining variables did not contribute significantly to the heterogeneity. Detailed results of the meta-regression analyses for all variables are provided in Supplementary Material 3.

### Mortality of sepsis

**Fig. 11:**
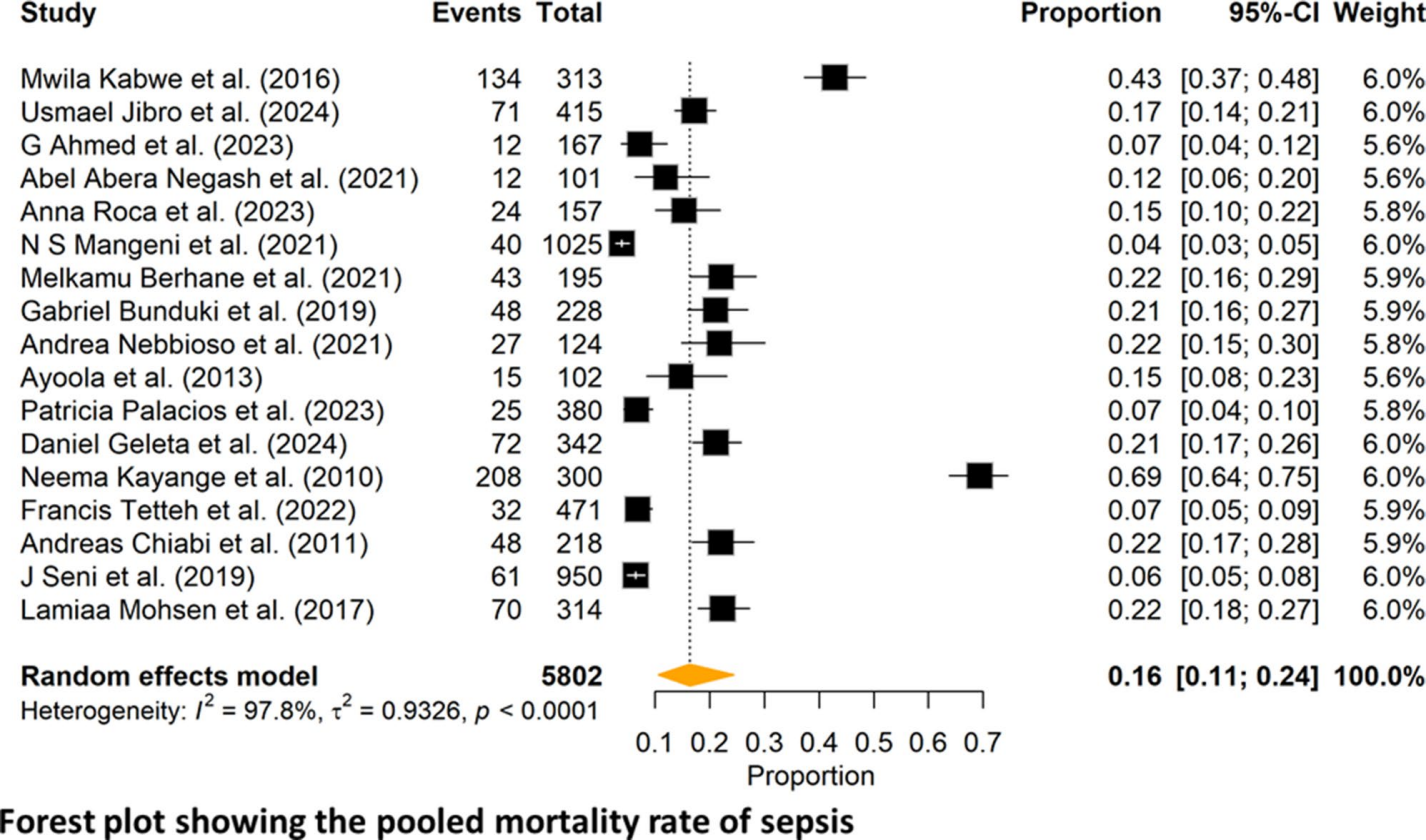
Forest plot showing the pooled mortality rate of sepsis

The mortality rate was estimated based on **17** studies that provided specific data on patient outcomes. The overall pooled mortality among clinically suspected sepsis cases was estimated to be 16.3% (95% CI: 10.6% to 24.4%) as shown in Fig. 11.

A very high level of heterogeneity was observed (*I*^2^ = 97.8%), as confirmed by a significant Cochran’s Q test (*p* < 0.001), indicating substantial variation in mortality estimates across studies. This variability is likely attributable to disparities in study design, clinical settings, case definitions, and population characteristics. The proportions across individual studies ranged widely, from 3.9% to 69.3% [52].

The presence of publication bias was assessed using Egger’s linear regression test for funnel plot asymmetry. The test did not indicate significant evidence of small-study effects *(t* = −1.76, *df* = 15, *p* = 0.099). Although the bias estimate was −10.02 (*SE* = 5.70), the result was not statistically significant at the conventional 0.05 threshold. Therefore, although the funnel plot may suggest asymmetry, there is no strong statistical evidence of publication bias in the mortality data.

### Factors associated with sepsis

**Fig. 12:**
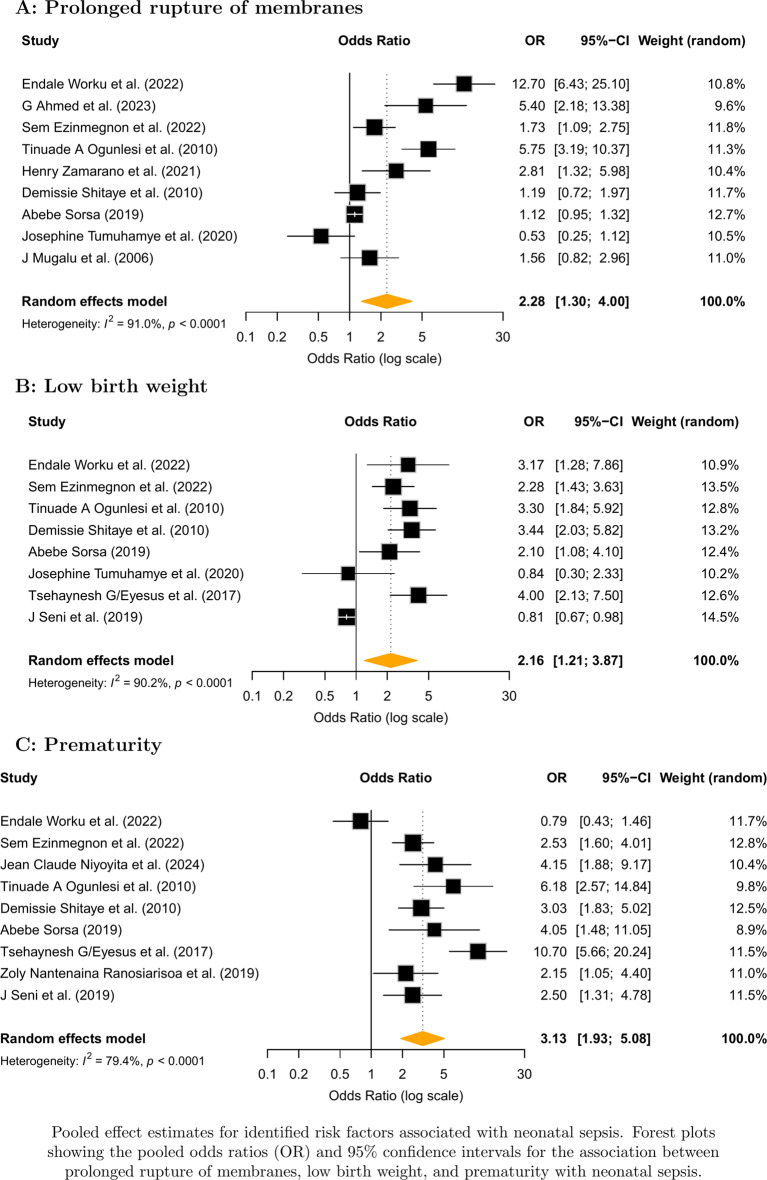
Pooled effect estimates for identified risk factors associated with neonatal sepsis. Forest plots showing the pooled odds ratios (OR) and 95% confidence intervals for the association between (**A**) prolonged rupture of membranes, (**B**) low birth weight, and (**C**) prematurity with neonatal sepsis

An analysis of factors associated with sepsis was conducted using data from studies that reported quantitative estimates. Several maternal and neonatal risk factors were identified as significant predictors of sepsis, as summarized in Fig. 12. Specifically, prolonged rupture of membranes was associated with a more than twofold increase in risk (OR = 2.28; 95% CI: 1.30 to 4.00; *p* < 0.001; *n* = 9 Studies). Similarly, low birth weight (OR = 2.16; 95% CI: 1.21 to 3.87, *p* < 0.001; *n* = 8 studies) and prematurity (OR = 3.13; 95% CI: 1.94 to 5.08, *p* < 0.001; *n* = 9 studies) were found to significantly increase the odds of developing sepsis.

Conversely, other clinical factors were not significantly associated with sepsis in this meta-analysis, including maternal fever (*p* = 0.180, *n* = 6 studies), an APGAR score less than 7 at five minutes (*p* = 0.885, *n* = 6 studies), instrumental vaginal delivery (*p* = 0.760, *n* = 4 studies), cesarean section (*p* = 0.502, *n* = 5 studies), and abnormal amniotic fluids (*p* = 0.226, *n* = 5 studies).

### Risk of bias in primary studies

The risk of bias was assessed using the National Heart, Lung, and Blood Institute’s (NHLBI) Quality Assessment Tool for Observational Cohort and Cross-Sectional Studies or Quality Assessment of Controlled Intervention Studies. Following a comprehensive evaluation of each item, the overall methodological quality of the included studies was satisfactory. All studies were rated as either good or fair, indicating a low to moderate risk of bias, and suggesting that the evidence base is generally reliable (Supplementary Material 4).

## Discussion

This systematic review and meta-analysis sought to evaluate the causative pathogens of sepsis in African children under five years of age. Specifically, the study aimed to delineate the distribution of these pathogens across African regions, analyze contextual variations, and provide robust estimates of the pooled prevalence and mortality of sepsis in this vulnerable population. Furthermore, the analysis identified key risk factors contributing significantly to the burden of neonatal sepsis.

### Etiological pathogens

A total of 59,521 pathogens were documented across the included studies, showing significant regional variation. *Klebsiella pneumoniae*, *Acinetobacter baumannii,* and *Staphylococcus aureus* emerged as the most prevalent pathogens. These findings align with previous reviews conducted in developing countries, confirming that *Staphylococcus aureus* and *Klebsiella* species remain the most prevalent pathogens causing neonatal sepsis across the African continent [66, 67]. This consistency is expected, as neonates represent 98% of our study population. Other frequently isolated pathogens included coagulase-negative staphylococci, *Enterococcus species*, *Escherichia coli*, and Group B *Streptococcus*. In contrast, fungal pathogens accounted for only 7.5% of cases, whereas viral pathogens were exceedingly rare (0.04%).

Our findings confirm that bacterial infections remain the overwhelming driver of sepsis in children under five, significantly contributing to regional morbidity and mortality [68]. Approximately 32% of neonatal deaths are attributable to infections, including septicemia, meningitis, respiratory infections, diarrhea, and neonatal tetanus [69].

Blood culture remains the gold standard for sepsis diagnosis [70–72]; however, its focus on bacterial growth inherently favors bacterial detection leading to a significant underestimation of non-bacterial etiologies [73]. Standard sepsis evaluations typically exclude viral panels, relegating them to secondary investigation only when bacterial, fungal, or parasitic sources are excluded [74]. This diagnostic delay is critical and potentially leads to unrecognized deaths in patients with viral sepsis. Although the emergence of viral-specific polymerase chain reaction assays and other advanced molecular tools has greatly enhanced viral diagnostic capabilities [74], these technologies remain largely inaccessible in low-income African contexts.

### Regional variations in pathogen distribution

Significant regional variation in pathogen distribution patterns was observed. Southern Africa accounted for the vast majority of isolates (*n* = 55,355/59,521). In contrast, reporting was considerably lower in Eastern (*n* = 1,925/59,521), Western (*n* = 1,440/59,521), Northern (*n* = 649/59,521), and Central Africa (*n* = 152/59,521), highlighting a critical gap in data collection and microbiological capacity in these regions.

The higher number of isolates reported from Southern Africa likely reflects differences in diagnostic capacity, laboratory infrastructure, and surveillance systems, rather than indicating a genuinely higher epidemiological burden of sepsis-causing pathogens. Importantly, the number of reported isolates does not necessarily reflect the true epidemiological burden of pathogens. Accurate estimation of pathogen burden would ideally contextualize isolate counts against a defined denominator related to covered target population, taking these underlying factors into account, to allow for appropriate interpretation of reported isolates across different African regions. Several operational and technical factors can influence the detection and reporting of pathogens. Advanced diagnostic tools, such as automated culture systems and multiplex molecular pathogen panels, enable the detection of a wider and more comprehensive range of pathogens. In contrast, limited diagnostic capacity can lead to underreporting, particularly of fastidious or less common organisms. Robust laboratory infrastructure, including well-equipped facilities, supports higher sample throughput. Additionally, established surveillance networks facilitate the systematic collection, aggregation, and reporting of microbiological data across multiple sites. Taken together, these factors highlight that reported isolate numbers should be interpreted with caution and not assumed to directly represent the true epidemiological burden.

Gram-negative bacteria predominated across most regions, with Southern Africa reporting the highest proportion, followed by Eastern and Northern Africa. In Southern Africa, *Klebsiella pneumoniae* and *Acinetobacter baumannii* were the primary isolates. Similarly, in Eastern Africa, *Klebsiella pneumoniae* and *Staphylococcus aureus* were predominant. Despite the overall Gram-negative trend in Central and Northern Africa, the most frequently reported individual species were *Staphylococcus aureus* and Coagulase-negative staphylococci, respectively.

Western Africa emerged as a unique outlier, being the only region in which Gram-positive bacteria were more frequent. This trend was largely driven by a high prevalence of *Staphylococcus aureus* and Coagulase-negative staphylococci. While fungal pathogens, particularly *Candida* species, were reported primarily in Southern Africa, they remained sporadic elsewhere. Viral pathogens were exceptionally rare, identified in only one study from Western Africa.

Overall, Southern Africa exhibits the highest number and diversity of isolates, characterized by Gram-negative dominance. In contrast, the prevalence of Gram-positive infections in Western Africa highlights critical regional variations. These distinct profiles underscore the necessity of localized surveillance and tailored antimicrobial stewardship strategies to effectively manage sepsis across the continent [75, 76].

### Prevalence, mortality, and risk factors

The pooled prevalence of sepsis was 40.2% (95% CI: 26.8% to 55.4%). This estimate aligns with previous reviews on neonatal sepsis in Africa, which reported prevalences of 40.98% (95% CI: 30.50% to 51.46%) [77], 36.02% (95% CI, 26.68 to 49.36) [78], and 38.56% (95% CI: 29.95% to 47.18%) [79]. In comparison, these figures are substantially higher than those found in Latin America, (26.46%, 95% CI: 8.60% to 44.33%) and Asia (14.68%, 95% CI: 9.39% to 19.97%) [79]. Underscoring a disproportionately high incidence within the African pediatric population.

The overall mortality rate was estimated at 16.3% (95% CI: 10.6%–24.4%), with individual studies ranging from 3.9% to 69.3%. This burden far exceeds the 1.3% to 5.4%, mortality rates reported in high and middle-income countries [80]. While these findings highlight a critical public health challenge, the pooled estimates should be interpreted with caution, as they reflect aggregated estimates rather than individual clinical risks. They may be influenced by variations in study populations, case definitions, and healthcare settings. Consequently, this high mortality should be interpreted with caution. The elevated burden of sepsis in Africa is multifactorial, stemming from a combination of structural and behavioral challenges. Key drivers include restricted access to high-quality healthcare, deficient pediatric infrastructure, and significant delays in seeking medical consultation. Furthermore, suboptimal hygiene protocols during labor and delivery remain critical contributors to the high incidence of infections in Africa [77].

Specific maternal and perinatal conditions significantly influence sepsis outcomes. Our results identify prolonged rupture of membranes, low birth weight (<2500 g), and preterm birth (gestational age < 37 weeks) as critical risk factors. While these results align with findings from previously conducted reviews [77, 78, 81], this study provides an important geographic scope and longitudinal temporal coverage by synthesizing evidence spanning more than two decades from across the entire African continent. Unlike many earlier reviews that focused primarily on Sub-Saharan Africa, this analysis demonstrates that these risk factors persist as dominant drivers of neonatal sepsis across diverse regions and healthcare settings.

The persistence of these associations in more recent studies suggests that traditional perinatal vulnerabilities remain insufficiently addressed despite evolving clinical guidelines and improvements in neonatal care. This consistency across regions also strengthens the evidence that these factors represent fundamental determinants of neonatal susceptibility to sepsis. These findings highlight the continued importance of strengthening preventive strategies during pregnancy, delivery, and the immediate postnatal period, particularly through improved intrapartum care and early neonatal monitoring.

Moreover, the sustained presence of these risk factors across regions raises important questions regarding potential underlying biological or pathogen-related mechanisms contributing to neonatal susceptibility. Future prospective studies including genetic, genomic and molecular epidemiological approaches could help determine whether host susceptibility, pathogen virulence characteristics, or the circulation of specific microbial sub-lineages that disproportionately affect neonates with these vulnerability profiles contribute to the observed patterns of neonatal sepsis in African settings. Understanding these mechanisms may provide important insights for developing more targeted prevention strategies and improving clinical management in high-risk neonatal populations.

### Methodological challenges and research gaps

Our review revealed substantial heterogeneity in sepsis definitions. While a minority of studies adopted internationally recognized standards such as the WHO guidelines or the International Pediatric Sepsis Consensus, the majority relied on empirical clinical signs. In many cases, explicit criteria were not specified. This variability is consistent with previous reports highlighting the lack of consensus in pediatric sepsis definitions worldwide, which pose a major obstacle to the accurate estimation of the global burden of sepsis [80, 82]. In African contexts, reliance on clinical diagnosis is often a necessity due to limited access to laboratory confirmation and biomarker testing [83]. Studies from sub-Saharan Africa illustrate how clinicians adapt definitions to resource-constrained environments by using simplified or locally tailored criteria [84, 85]. A recent survey across 25 African countries confirmed this trend, showing that diagnostic practices for neonatal and pediatric sepsis range widely, and are often left to clinician judgment, reflecting a lack of harmonized standards [86].

Such inconsistency likely contributed to the high heterogeneity in prevalence observed in our meta-analysis. Furthermore, it limits the comparability of findings across different studies and healthcare settings. The harmonization of diagnostic criteria, ideally through resource-sensitive and context-adapted guidelines, is essential. Strengthening these standards will improve surveillance, refine empirical treatment strategies, and enable reliable regional and global comparisons.

The strong emphasis on the neonatal period in our review underscores its critical importance, driven by the heightened biological vulnerability of newborns and the disproportionate sepsis-related mortality in newborns. However, our findings reveal a notable paucity of studies targeting the post-neonatal period, infants, and children up to five years of age. This research gap suggests that the epidemiology, etiology, and outcomes of sepsis in older infants and preschool children remain insufficiently characterized. This imbalance limits a comprehensive understanding of the full pediatric sepsis spectrum. Addressing this disparity must be a priority for future research to ensure that empirical treatment guidelines and health policies adequately serve the entire under-five population.

### Strengths and limitations

#### Strengths

This systematic review possesses several methodological strengths. It was conducted in strict accordance with PRISMA guidelines and follows a pre-registered PROSPERO protocol. The study involved an extensive literature search across major databases, encompassing data from 19 African nations. Furthermore, the substantial cumulative sample size of over 98,000 patients significantly enhances the representativeness of the findings. The application of robust statistical techniques, including the trim-and-fill method to adjust for publication bias, ensures the analytical rigor of our results.

#### Limitations

Several limitations must be acknowledged. First, substantial heterogeneity (*I*^2^ > 97%) was observed, which may constrain the precision of the pooled estimates. Second, the majority of included studies were observational and often retrospective, and many lacked explicit sample size calculations, which may compromise internal validity. Third, as data were primarily derived from hospital-based settings, the findings may not be fully generalizable to community-level populations. Additionally, the sparse reporting of viral and fungal pathogens, inconsistent clinical definitions of sepsis, and the limited representation of the post-neonatal period (up to five years of age) limit the comprehensiveness of our etiological conclusions. Finally, although age-based subgroup analyses were pre-specified, inconsistent reporting across studies precluded reliable age-stratified assessments.

### Implications of the findings

#### Clinical implications

The predominance of *Klebsiella pneumoniae*, *Acinetobacter baumannii*, and *Staphylococcus aureus*, coupled with high regional variations, underscores that “one-size-fits-all” empirical antibiotic protocols are insufficient. Treatment must be tailored to local resistance and pathogen profiles while awaiting culture results. Identifying premature rupture of membranes, prematurity, and low birth weight as primary risk factors allows clinicians to implement intensified monitoring and early intervention strategies for these high-risk neonates.

#### Public health research implications

The substantial burden of sepsis in Africa, which significantly exceeds rates in other developing regions, highlights a critical need for enhanced surveillance and prevention programs. The observed regional variations in pathogen profiles suggest that a “one-size-fits-all” approach is inadequate. Instead, public health initiatives should focus on localized interventions, such as improving maternal hygiene, promoting safe delivery practices, and strengthening local laboratory diagnostic capacity.

Furthermore, the significant under-detection of viral and fungal pathogens represents a critical “blind spot” in current surveillance. Integrating molecular tools, such as PCR, into standard diagnostic protocols is vital for accurately assessing the true burden of non-bacterial sepsis and refining therapeutic responses.

Finally, the paucity of data on children aged 1 month to 5 years underscores the urgent need for public health programs to expand their focus beyond the immediate neonatal period. By encompassing the full spectrum of pediatric mortality, health authorities can ensure that surveillance and prevention strategies adequately protect the entire under-five population.

#### Policy implications

Policymakers must advocate for harmonized, resource-sensitive diagnostic criteria. Standardizing sepsis definitions across Africa is the only way to ensure reliable data comparison and effective resource allocation.

## Conclusion

This systematic review and meta-analysis provides a comprehensive overview of the causative pathogens, prevalence, mortality, and associated risk factors for sepsis among children under five years old of age in Africa. Our findings identify bacterial infections, specifically Gram-negative bacteria such as *Klebsiella pneumoniae* and *Acinetobacter baumannii*, alongside *Staphylococcus aureus*, as the primary drivers of the sepsis burden, with significant regional variations across the continent. The high pooled prevalence (40%, 95% CI: 26.8% to 55.4%) and mortality rate (16.3%, 95% CI: 10.6%–24.4%) emphasize the substantial burden of sepsis in African pediatric populations, especially within hospital settings. The identified risk factors related mainly to maternal and perinatal conditions suggest key targets for early intervention and improved neonatal care. The findings underscore the urgent need to enhance sepsis surveillance, diagnosis, and management in Africa, and to tailor prevention and treatment strategies according to local pathogen profiles.

## Electronic supplementary material

Below is the link to the electronic supplementary material.

Supplementary Material 1: Detailed search strategies used for PubMed, Web of Sciences and Scopus database

Supplementary Material 2: Adjusted meta-analytic methods Trim-and-Fill procedure

Supplementary Material 3: Meta régression plots

Supplementary Material 4: Study Quality Assessment Tools

## Data Availability

Epi info template data collection forms and extracted data from the original included studies are available upon reasonable request from the corresponding author.

## Abbreviations

*CI*: Confidence Interval
*OR*: Odds Ratio
