# Supplementary material for: Etiology, prevalence, and mortality of sepsis among children under five years in Africa: a systematic review and meta-analysis
Source: BMC Infect Dis. 2026 Apr 25;26:1121. doi: 10.1186/s12879-026-13350-2 (PMC13255319; doi:10.1186/s12879-026-13350-2)
Supplement: Supplementary file 1 — Supplementary Material 1: Detailed search strategies used for PubMed, Web of Sciences and Scopus database [file 12879_2026_13350_MOESM1_ESM.docx]

**Supplementary Material 1:** Detailed search strategies.

- **PubMed**

| Search | Query | Results |
| --- | --- | --- |
| #1 | **"Sepsis"[Title/Abstract] OR "septicem*"[Title/Abstract] OR "septicaem*"[Title/Abstract] OR "septic shock"[Title/Abstract]** | [170,456](https://pubmed.ncbi.nlm.nih.gov/?term=%22Sepsis%22%5BTitle%2FAbstract%5D+OR+%22septicem%2A%22%5BTitle%2FAbstract%5D+OR+%22septicaem%2A%22%5BTitle%2FAbstract%5D+OR+%22septic+shock%22%5BTitle%2FAbstract%5D&sort=) |
| #2 | **"Prevalence"[Title/Abstract] OR "etiolog*"[Title/Abstract] OR "aetiolog*"[Title/Abstract] OR "outcom*"[Title/Abstract] OR "Mortality"[Title/Abstract]** | [4,479,812](https://pubmed.ncbi.nlm.nih.gov/?term=%22Prevalence%22%5BTitle%2FAbstract%5D+OR+%22etiolog%2A%22%5BTitle%2FAbstract%5D+OR+%22aetiolog%2A%22%5BTitle%2FAbstract%5D+OR+%22outcom%2A%22%5BTitle%2FAbstract%5D+OR+%22Mortality%22%5BTitle%2FAbstract%5D&sort=) |
| #3 | **"Angola"[Title/Abstract] OR "Algeria"[Title/Abstract] OR "Benin"[Title/Abstract] OR "Botswana"[Title/Abstract] OR "Burkina Faso"[Title/Abstract] OR "Burundi"[Title/Abstract] OR "Cameroon"[Title/Abstract] OR "Cape Verde"[Title/Abstract] OR "Central African Republic"[Title/Abstract] OR "Chad"[Title/Abstract] OR "Comoros"[Title/Abstract] OR "Republic of the Congo"[Title/Abstract] OR "Congo Brazzaville"[Title/Abstract] OR "Democratic republic of the Congo"[Title/Abstract] OR "Cote d'Ivoire"[Title/Abstract] OR "Djibouti"[Title/Abstract] OR "Equatorial Guinea"[Title/Abstract] OR "Egypt"[Title/Abstract] OR "Eritrea"[Title/Abstract] OR "Ethiopia"[Title/Abstract] OR "Gabon"[Title/Abstract] OR "The Gambia"[Title/Abstract] OR "Ghana"[Title/Abstract] OR "Guinea"[Title/Abstract] OR "Guinea-Bissau"[Title/Abstract] OR "Kenya"[Title/Abstract] OR "Libye"[Title/Abstract] OR "Lesotho"[Title/Abstract] OR "Liberia"[Title/Abstract] OR "Madagascar"[Title/Abstract] OR "Malawi"[Title/Abstract] OR "Mali"[Title/Abstract] OR "Mauritania"[Title/Abstract] OR "Mauritius"[Title/Abstract] OR "Morocco"[Title/Abstract] OR "Mozambique"[Title/Abstract] OR "Namibia"[Title/Abstract] OR "Niger"[Title/Abstract] OR "Nigeria"[Title/Abstract] OR "Reunion"[Title/Abstract] OR "Rwanda"[Title/Abstract] OR "Sao Tome and Principe"[Title/Abstract] OR "Senegal"[Title/Abstract] OR "Seychelles"[Title/Abstract] OR "Sierra Leone"[Title/Abstract] OR "Somalia"[Title/Abstract] OR "South Africa"[Title/Abstract] OR "Sudan"[Title/Abstract] OR "Swaziland"[Title/Abstract] OR "Eswatini"[Title/Abstract] OR "Tanzania"[Title/Abstract] OR "Togo"[Title/Abstract] OR "Tunisia"[Title/Abstract] OR "Uganda"[Title/Abstract] OR "Western Sahara"[Title/Abstract] OR "Zambia"[Title/Abstract] OR "Zimbabwe"[Title/Abstract]** | [453,231](https://pubmed.ncbi.nlm.nih.gov/?term=longquerybd7a60100257b2a56955&sort=) |
| #4 | **((#1) AND (#2) AND (#3))** | [2,004](https://pubmed.ncbi.nlm.nih.gov/?term=%28%28%231%29+AND+%28%232%29+AND+%28%233%29%29&sort=) |
| #5 | **((#4) NOT (Review [Publication Type] OR "Systematic Review"[Publication Type] OR "Meta-analysis"[Publication Type]))** | [1,936](https://pubmed.ncbi.nlm.nih.gov/?term=%28%234%29+NOT+%28Review+%5BPublication+Type%5D+OR+%22Systematic+Review%22%5BPublication+Type%5D+OR+%22Meta-analysis%22%5BPublication+Type%5D%29&sort=) |
| #6 | #5 and Filters: **Newborn: birth-1 month, Infant: birth-23 months, Infant: 1-23 months, Preschool Child: 2-5 years** | [743](https://pubmed.ncbi.nlm.nih.gov/?term=%28%234%29+NOT+%28Review+%5BPublication+Type%5D+OR+%22Systematic+Review%22%5BPublication+Type%5D+OR+%22Meta-analysis%22%5BPublication+Type%5D%29&filter=age.newborn&filter=age.allinfant&filter=age.infant&filter=age.preschoolchild&sort=relevance) |
| #7 | #6 and Filters: **Humans,** | [742](https://pubmed.ncbi.nlm.nih.gov/?term=%28%234%29+NOT+%28Review+%5BPublication+Type%5D+OR+%22Systematic+Review%22%5BPublication+Type%5D+OR+%22Meta-analysis%22%5BPublication+Type%5D%29&filter=hum_ani.humans&filter=age.newborn&filter=age.allinfant&filter=age.infant&filter=age.preschoolchild&sort=relevance) |
| #8 | #6 and Filters: **English, French,** | [741](https://pubmed.ncbi.nlm.nih.gov/?term=%28%234%29+NOT+%28Review+%5BPublication+Type%5D+OR+%22Systematic+Review%22%5BPublication+Type%5D+OR+%22Meta-analysis%22%5BPublication+Type%5D%29&filter=lang.english&filter=lang.french&filter=hum_ani.humans&filter=age.newborn&filter=age.allinfant&filter=age.infant&filter=age.preschoolchild&sort=relevance) |
| #9 | Filters**: from 2000 - 2024** | [655](https://pubmed.ncbi.nlm.nih.gov/?term=%28%234%29+NOT+%28Review+%5BPublication+Type%5D+OR+%22Systematic+Review%22%5BPublication+Type%5D+OR+%22Meta-analysis%22%5BPublication+Type%5D%29&filter=lang.english&filter=lang.french&filter=hum_ani.humans&filter=age.newborn&filter=age.allinfant&filter=age.infant&filter=age.preschoolchild&filter=years.2000-2025&sort=relevance) |
|  | ((("Sepsis"[Title/Abstract] OR "septicem*"[Title/Abstract] OR "septicaem*"[Title/Abstract] OR "septic shock"[Title/Abstract]) AND ("Prevalence"[Title/Abstract] OR "etiolog*"[Title/Abstract] OR "aetiolog*"[Title/Abstract] OR "outcom*"[Title/Abstract] OR "Mortality"[Title/Abstract]) AND ("Angola"[Title/Abstract] OR "Algeria"[Title/Abstract] OR "Benin"[Title/Abstract] OR "Botswana"[Title/Abstract] OR "Burkina Faso"[Title/Abstract] OR "Burundi"[Title/Abstract] OR "Cameroon"[Title/Abstract] OR "Cape Verde"[Title/Abstract] OR "Central African Republic"[Title/Abstract] OR "Chad"[Title/Abstract] OR "Comoros"[Title/Abstract] OR "Republic of the Congo"[Title/Abstract] OR "Congo Brazzaville"[Title/Abstract] OR "Democratic republic of the Congo"[Title/Abstract] OR "Cote d'Ivoire"[Title/Abstract] OR "Djibouti"[Title/Abstract] OR "Equatorial Guinea"[Title/Abstract] OR "Egypt"[Title/Abstract] OR "Eritrea"[Title/Abstract] OR "Ethiopia"[Title/Abstract] OR "Gabon"[Title/Abstract] OR "The Gambia"[Title/Abstract] OR "Ghana"[Title/Abstract] OR "Guinea"[Title/Abstract] OR "Guinea-Bissau"[Title/Abstract] OR "Kenya"[Title/Abstract] OR "Libye"[Title/Abstract] OR "Lesotho"[Title/Abstract] OR "Liberia"[Title/Abstract] OR "Madagascar"[Title/Abstract] OR "Malawi"[Title/Abstract] OR "Mali"[Title/Abstract] OR "Mauritania"[Title/Abstract] OR "Mauritius"[Title/Abstract] OR "Morocco"[Title/Abstract] OR "Mozambique"[Title/Abstract] OR "Namibia"[Title/Abstract] OR "Niger"[Title/Abstract] OR "Nigeria"[Title/Abstract] OR "Reunion"[Title/Abstract] OR "Rwanda"[Title/Abstract] OR "Sao Tome and Principe"[Title/Abstract] OR "Senegal"[Title/Abstract] OR "Seychelles"[Title/Abstract] OR "Sierra Leone"[Title/Abstract] OR "Somalia"[Title/Abstract] OR "South Africa"[Title/Abstract] OR "Sudan"[Title/Abstract] OR "Swaziland"[Title/Abstract] OR "Eswatini"[Title/Abstract] OR "Tanzania"[Title/Abstract] OR "Togo"[Title/Abstract] OR "Tunisia"[Title/Abstract] OR "Uganda"[Title/Abstract] OR "Western Sahara"[Title/Abstract] OR "Zambia"[Title/Abstract] OR "Zimbabwe"[Title/Abstract])) NOT ("Review"[Publication Type] OR "Systematic Review"[Publication Type] OR "Meta-analysis"[Publication Type])) AND ((humans[Filter]) AND (english[Filter] OR french[Filter]) AND (newborn[Filter] OR allinfant[Filter] OR infant[Filter] OR preschoolchild[Filter]) AND (2000:2024[pdat])) | [655](https://pubmed.ncbi.nlm.nih.gov/?term=%28%234%29+NOT+%28Review+%5BPublication+Type%5D+OR+%22Systematic+Review%22%5BPublication+Type%5D+OR+%22Meta-analysis%22%5BPublication+Type%5D%29&filter=lang.english&filter=lang.french&filter=hum_ani.humans&filter=age.newborn&filter=age.allinfant&filter=age.infant&filter=age.preschoolchild&filter=years.2000-2025&sort=relevance) |

- **Web of Sciences core collection**

| Search | Query | Results |
| --- | --- | --- |
| #1 | **TS=(Sepsis OR septicem* OR septicaem* OR "Septic Shock")** | [131,544](https://www.webofscience.com/wos/woscc/summary/72f50b29-8993-4d16-8a6e-26aa5f11b232-013f4ee7ad/relevance/1) |
| #2 | **TS=(Angola OR Algeria OR Benin OR Botswana OR "Burkina Faso" OR Burundi OR Cameroon OR "Cape Verde" OR "Central African Republic" OR Chad OR Comoros OR "Republic of the Congo" OR "Congo Brazzaville" OR "Democratic republic of the Congo" OR "Cote d’Ivoire" OR Djibouti OR "Equatorial Guinea" OR Egypt OR Eritrea OR Ethiopia OR Gabon OR "The Gambia" OR Ghana OR Guinea OR Guinea-Bissau OR Kenya OR Libye OR Lesotho OR Liberia OR Madagascar OR Malawi OR Mali OR Mauritania OR Mauritius OR Morocco OR Mozambique OR Namibia OR Niger OR Nigeria OR Reunion OR Rwanda OR "Sao Tome and Principe" OR Senegal OR Seychelles OR "Sierra Leone" OR Somalia OR "South Africa" OR Sudan OR Swaziland OR Eswatini OR Tanzania OR Togo OR Tunisia OR Uganda OR "Western Sahara" OR Zambia OR Zimbabwe)** |  |
| #3 | **TS=(Prevalence OR Etiolog* OR Aetiolog* OR Outcom* OR Mortality)** | [4,584,281](https://www.webofscience.com/wos/woscc/summary/b437dbe5-5dee-4db6-818a-2b0568ddb34c-013f4f5e31/relevance/1) |
| #4 | **TS=(Newborn OR neonate OR Infant OR ''Preschool Child'')** | [444,570](https://www.webofscience.com/wos/woscc/summary/f89813f7-b270-463a-b998-6b5bd0556c33-013f4f7ebb/relevance/1) |
| #5 | **#4 AND #3 AND #2 AND #1** | [676](https://www.webofscience.com/wos/woscc/summary/1184646a-e0b9-420a-97b2-f0054e69608c-013f4f938c/relevance/1) |
| #6 | **#1 AND #2 AND #3 AND #4** and **Review Article** or **Editorial Material** or **Proceeding Paper** or **Letter** or **Meeting Abstract** (Exclude – Document Types) | [626](https://www.webofscience.com/wos/woscc/summary/92f042d7-68b5-48f7-a899-f9435c6c7b33-013f4fb91d/relevance/1) |
| #7 | **#1 AND #2 AND #3 AND #4** and **Review Article** or **Editorial Material** or **Proceeding Paper** or **Letter** or **Meeting Abstract** (Exclude – Document Types) and **English** or **French** (Languages) | [625](https://www.webofscience.com/wos/woscc/summary/6c97730f-01d7-4f25-8fdc-1032a20486a3-013f4fe3fc/relevance/1) |
| #8 |  |  |

- **Scopus**

| Search | Query | Results |
| --- | --- | --- |
| #1 | TITLE-ABS-KEY ( sepsis OR septicem* OR septicaem* OR "septic shock ") | 318,043 |
| #2 | TITLE-ABS-KEY ( angola OR algeria OR benin OR botswana OR "burkina faso" OR burundi OR cameroon OR "cape verde" OR "central african republic" OR chad OR comoros OR "republic of the congo" OR "congo brazzaville" OR "democratic republic of the congo" OR djibouti OR "equatorial guinea" OR egypt OR eritrea OR ethiopia OR gabon OR "the gambia" OR ghana OR guinea OR guinea-bissau OR kenya OR libye OR lesotho OR liberia OR madagascar OR malawi OR mali OR mauritania OR mauritius OR morocco OR mozambique OR namibia OR niger OR nigeria OR reunion OR rwanda OR "sao tome and principe" OR senegal OR seychelles OR "sierra leone" OR somalia OR "south africa" OR sudan OR swaziland OR eswatini OR tanzania OR togo OR tunisia OR uganda OR "western sahara" OR zambia OR zimbabwe ) | 1,305,703 |
| #3 | TITLE-ABS-KEY ( prevalence OR etiolog* OR aetiolog* OR outcom* OR mortality ) | 8,135,616 |
| #4 | #1 AND #2 AND #3 | 3,949 |
| #5 | #4  AND ( EXCLUDE ( EXACTKEYWORD , "Adult" ) OR EXCLUDE ( EXACTKEYWORD , "Adolescent" ) OR EXCLUDE ( EXACTKEYWORD , "Young Adult" ) OR EXCLUDE ( EXACTKEYWORD , "Aged" ) OR EXCLUDE ( EXACTKEYWORD , "Age" ) OR EXCLUDE ( EXACTKEYWORD , "Aged, 80 And Over" ) OR EXCLUDE ( EXACTKEYWORD , "Middle Aged" ) ) | 1,415 |
| #6 | #5  AND ( EXCLUDE ( DOCTYPE , "re" ) OR EXCLUDE ( DOCTYPE , "le" ) OR EXCLUDE ( DOCTYPE , "no" ) OR EXCLUDE ( DOCTYPE , "ed" ) OR EXCLUDE ( DOCTYPE , "cp" ) OR EXCLUDE ( DOCTYPE , "ch" ) OR EXCLUDE ( DOCTYPE , "sh" ) OR EXCLUDE ( DOCTYPE , "bk" ) ) | 1,208 |
| #7 | #6 AND PUBYEAR > 1999 AND PUBYEAR < 2025 AND | 1002 |
| #8 | #7 AND LIMIT-TO ( EXACTKEYWORD , "Human" ) | 666 |
|  | (TITLE-ABS-KEY ( angola OR algeria OR benin OR botswana OR "burkina faso" OR burundi OR cameroon OR "cape verde" OR "central african republic" OR chad OR comoros OR "republic of the congo" OR "congo brazzaville" OR "democratic republic of the congo" OR djibouti OR "equatorial guinea" OR egypt OR eritrea OR ethiopia OR gabon OR "the gambia" OR ghana OR guinea OR guinea-bissau OR kenya OR libye OR lesotho OR liberia OR madagascar OR malawi OR mali OR mauritania OR mauritius OR morocco OR mozambique OR namibia OR niger OR nigeria OR reunion OR rwanda OR "sao tome and principe" OR senegal OR seychelles OR "sierra leone" OR somalia OR "south africa" OR sudan OR swaziland OR eswatini OR tanzania OR togo OR tunisia OR uganda OR "western sahara" OR zambia OR zimbabwe )) AND (TITLE-ABS-KEY ( prevalence OR etiolog* OR aetiolog* OR outcom* OR mortality )) AND (TITLE-ABS-KEY ( sepsis OR septicem* OR septicaem* OR "septic shock " )) AND PUBYEAR > 1999 AND PUBYEAR < 2025 AND ( EXCLUDE ( EXACTKEYWORD,"Adult" ) OR EXCLUDE ( EXACTKEYWORD,"Adolescent" ) OR EXCLUDE ( EXACTKEYWORD,"Young Adult" ) OR EXCLUDE ( EXACTKEYWORD,"Aged" ) OR EXCLUDE ( EXACTKEYWORD,"Age" ) OR EXCLUDE ( EXACTKEYWORD,"Aged, 80 And Over" ) OR EXCLUDE ( EXACTKEYWORD,"Middle Aged" ) OR LIMIT-TO ( EXACTKEYWORD,"Human" ) ) AND ( EXCLUDE ( DOCTYPE,"re" ) OR EXCLUDE ( DOCTYPE,"le" ) OR EXCLUDE ( DOCTYPE,"no" ) OR EXCLUDE ( DOCTYPE,"ed" ) OR EXCLUDE ( DOCTYPE,"cp" ) OR EXCLUDE ( DOCTYPE,"ch" ) OR EXCLUDE ( DOCTYPE,"sh" ) OR EXCLUDE ( DOCTYPE,"bk" ) ) | 666 |
