# Supplementary material for: Etiology, prevalence, and mortality of sepsis among children under five years in Africa: a systematic review and meta-analysis
Source: BMC Infect Dis. 2026 Apr 25;26:1121. doi: 10.1186/s12879-026-13350-2 (PMC13255319; doi:10.1186/s12879-026-13350-2)
Supplement: Supplementary file 2 — Supplementary Material 2: Adjusted meta-analytic methods Trim-and-Fill procedure [file 12879_2026_13350_MOESM2_ESM.docx]

**Supplementary Material 2: Adjusted meta-analyse methods ‘’Trim-and-Fill procedure’’ results**

Study Prevalence [95%-CI] %W(random)

Mwila Kabwe et al.2016 0.3296 [0.2798; 0.3835] 1.9

Dharshni Pillay et al.2021 0.9993 [0.9884; 1.0000] 1.3

Usmael Jibro et al.2024 0.0517 [0.0341; 0.0776] 1.8

Minichil Worku et al.2022 0.5000 [0.4385; 0.5615] 1.9

Endale Worku et al.2022 0.3651 [0.3190; 0.4139] 1.9

Rudzani Mashau et al.2022 1.0000 [0.9998; 1.0000] 1.3

G Ahmed et al.2023 0.0982 [0.0615; 0.1533] 1.8

Gashaw Amsalu et al.2024 0.2170 [0.1780; 0.2618] 1.9

Abel Abera et al.2021 0.3382 [0.2532; 0.4351] 1.8

Sem Ezinmegnon et al.2022 0.1864 [0.1568; 0.2202] 1.9

Anna Roca et al.2023 0.5601 [0.4819; 0.6355] 1.9

Reenu Thomas et al.2024 0.2656 [0.2612; 0.2700] 1.9

N S Mangeni et al.2021 0.1623 [0.1410; 0.1861] 1.9

Guy Mulinganya et al.2021 0.4073 [0.3318; 0.4874] 1.9

Francis Wuni et al.2023 0.1354 [0.0999; 0.1809] 1.9

Jean Niyoyita et al.2024 0.0508 [0.0335; 0.0763] 1.8

Tinuade Ogunlesi et al.2010 0.3305 [0.2917; 0.3718] 1.9

Melkamu Berhane et al.2021 0.5893 [0.5191; 0.6560] 1.9

Gabriel Bunduki et al.2019 0.3035 [0.2474; 0.3661] 1.9

Andrea Nebbioso et al.2021 0.2680 [0.1977; 0.3523] 1.8

Henry Zamarano et al.2021 0.5894 [0.5006; 0.6728] 1.9

Demissie Shitaye et al.2010 0.4472 [0.3921; 0.5036] 1.9

Abebe Sorsa 2019 0.2911 [0.2428; 0.3447] 1.9

Ruchika Kohli et al.2011 0.2290 [0.1986; 0.2624] 1.9

Mulat Dagnew et al.2013 0.3308 [0.2277; 0.4531] 1.8

Ayoola et al.2013 0.3835 [0.2949; 0.4806] 1.8

Kenneth Iregbu et al.2006 0.2187 [0.1805; 0.2624] 1.9

Martin Meremikwu et al.2005 0.4761 [0.4459; 0.5064] 1.9

W.A. Seliem et al.2018 0.3466 [0.2822; 0.4171] 1.9

Fortress Aku et al.2020 0.1755 [0.1228; 0.2446] 1.8

E.A. Adejuyigbe et al.2001 0.5542 [0.4645; 0.6405] 1.9

Josephine Tumuhamye et al.2020 0.1292 [0.0983; 0.1679] 1.9

Patricia Palacios et al.2023 0.5236 [0.4734; 0.5734] 1.9

Daniel Geleta et al.2024 0.4038 [0.3531; 0.4566] 1.9

Tsehaynesh Eyesus et al.2017 0.4663 [0.4055; 0.5281] 1.9

Neema Kayange et al.2010 0.4967 [0.4405; 0.5530] 1.9

J Mugalu et al.2006 0.3759 [0.3223; 0.4326] 1.9

Eman Shehab et al.2015 0.3925 [0.3432; 0.4440] 1.9

Pius Simon et al.2016 0.4189 [0.3309; 0.5125] 1.9

Zoly Ranosiarisoa et al.2019 0.4302 [0.3760; 0.4861] 1.9

Lydia Mudzikatib et al.2015 0.2709 [0.2430; 0.3007] 1.9

Francis Tetteh et al.2022 0.3252 [0.2844; 0.3688] 1.9

Andreas Chiabi et al.2011 0.2215 [0.1713; 0.2813] 1.9

J Seni et al.2019 0.1425 [0.1217; 0.1662] 1.9

Lamiaa Mohsen et al.2017 0.7794 [0.7302; 0.8218] 1.9

A O Mokuolu et al.2002 0.3090 [0.2488; 0.3766] 1.9

Filled: Minichil Worku et al.2022 0.1243 [0.0997; 0.1538] 1.9

Filled: Patricia Palacios et al.2023 0.1143 [0.0955; 0.1363] 1.9

Filled: E.A. Adejuyigbe et al.2001 0.1024 [0.0738; 0.1406] 1.9

Filled: Anna Roca et al.2023 0.1002 [0.0753; 0.1324] 1.9

Filled: Melkamu Berhane et al.2021 0.0900 [0.0692; 0.1162] 1.9

Filled: Henry Zamarano et al.2021 0.0899 [0.0645; 0.1240] 1.9

Filled: Lamiaa Mohsen et al.2017 0.0386 [0.0299; 0.0498] 1.9

Filled: Dharshni Pillay et al.2021 0.0001 [0.0000; 0.0017] 1.3

Filled: Rudzani Mashau et al.2022 0.0000 [0.0000; 0.0000] 1.3

Number of studies: k = 55 (with 9 added studies)

proportion 95%-CI

Random effects model 0.2713 [0.1551; 0.4302]

Quantifying heterogeneity (with 95%-CIs):

tau^2 = 5.0504; tau = 2.2473; I^2 = 97.9% [97.6%; 98.1%]; H = 6.88 [6.47; 7.32]

Test of heterogeneity:

Q d.f. p-value

2558.71 54 0

Details of meta-analysis methods:

- Inverse variance method

- Maximum-likelihood estimator for tau^2

- Calculation of I^2 based on Q

- Hartung-Knapp adjustment for random effects model (df = 54)

- Trim-and-fill method to adjust for funnel plot asymmetry (L-estimator)

- Logit transformation
