# Supplementary material for: Etiology, prevalence, and mortality of sepsis among children under five years in Africa: a systematic review and meta-analysis
Source: BMC Infect Dis. 2026 Apr 25;26:1121. doi: 10.1186/s12879-026-13350-2 (PMC13255319; doi:10.1186/s12879-026-13350-2)
Supplement: Supplementary file 3 — Supplementary Material 3: Meta régression plots [file 12879_2026_13350_MOESM3_ESM.docx]

### **Supplementary Material 3 : Meta-regression analysis results.**

*Figure 1. Meta-regression plot assessing the association between year of publication and prevalence of sepsis (no significant effect observed)*

*The following output summarizes the results from the mixed-effects meta-regression model using year of publication as a continuous moderator (method = REML).*

Mixed-Effects Model (k = 46; tau^2 estimator: REML)

logLik deviance AIC BIC AICc

6.2359 -12.4719 -6.4719 -1.1193 -5.8719

tau^2 (estimated amount of residual heterogeneity): 0.0437 (SE = 0.0095)

tau (square root of estimated tau^2 value): 0.2091

I^2 (residual heterogeneity / unaccounted variability): 99.93%

H^2 (unaccounted variability / sampling variability): 1503.27

R^2 (amount of heterogeneity accounted for): 0.00%

Test for Residual Heterogeneity:

QE(df = 44) = 158018.1907, p-val < .0001

Test of Moderators (coefficient 2):

QM(df = 1) = 0.2123, p-val = 0.6450

Model Results:

estimate se zval pval ci.lb ci.ub

intrcpt 5.0174 10.0944 0.4970 0.6192 -14.7673 24.8020

Year -0.0023 0.0050 -0.4608 0.6450 -0.0121 0.0075

---

Signif. codes: 0 ‘***’ 0.001 ‘**’ 0.01 ‘*’ 0.05 ‘.’ 0.1 ‘ ’ 1

*Figure 2. Meta-regression assessing the association between study design and prevalence of sepsis (no significant effect observed)*

*The following output summarizes the results from the mixed-effects meta-regression model using study design as a continuous moderator (method = REML).*

Mixed-Effects Model (k = 46; tau^2 estimator: REML)

logLik deviance AIC BIC AICc

4.3580 -8.7160 13.2840 30.7027 24.2840

tau^2 (estimated amount of residual heterogeneity): 0.0456 (SE = 0.0110)

tau (square root of estimated tau^2 value): 0.2135

I^2 (residual heterogeneity / unaccounted variability): 99.79%

H^2 (unaccounted variability / sampling variability): 476.92

R^2 (amount of heterogeneity accounted for): 0.00%

Test for Residual Heterogeneity:

QE(df = 36) = 93089.3042, p-val < .0001

Test of Moderators (coefficients 2:10):

QM(df = 9) = 6.3212, p-val = 0.7074

Model Results:

estimate se zval pval

intrcpt 0.4330 0.1242 3.4876 0.0005

Study_designCohort 0.0630 0.1968 0.3201 0.7489

Study_designCross sectional -0.1134 0.1417 -0.8008 0.4232

Study_designProspective -0.0130 0.1459 -0.0890 0.9291

Study_designProspective Cohort -0.2765 0.1966 -1.4061 0.1597

Study_designProspective Cross sectional -0.0798 0.1486 -0.5373 0.5911

Study_designProspective observational -0.3372 0.2482 -1.3587 0.1742

Study_designRandomized Controlled Trial 0.1275 0.2504 0.5092 0.6106

Study_designRetrospective -0.0531 0.1455 -0.3650 0.7151

Study_designRetrospective Cross sectional -0.0532 0.1645 -0.3233 0.7465

ci.lb ci.ub

intrcpt 0.1897 0.6764 ***

Study_designCohort -0.3227 0.4487

Study_designCross sectional -0.3911 0.1642

Study_designProspective -0.2989 0.2729

Study_designProspective Cohort -0.6619 0.1089

Study_designProspective Cross sectional -0.3710 0.2114

Study_designProspective observational -0.8237 0.1492

Study_designRandomized Controlled Trial -0.3632 0.6182

Study_designRetrospective -0.3382 0.2320

Study_designRetrospective Cross sectional -0.3757 0.2693

---

Signif. codes: 0 ‘***’ 0.001 ‘**’ 0.01 ‘*’ 0.05 ‘.’ 0.1 ‘ ’ 1

*Figure 3. Meta-regression plot evaluating the association between geographic region and prevalence of sepsis (no significant effect observed)*

*The following output summarizes the results from the mixed-effects meta-regression model using geographic region as a continuous moderator (method = REML).*

Mixed-Effects Model (k = 46; tau^2 estimator: REML)

logLik deviance AIC BIC AICc

7.6984 -15.3968 -3.3968 6.8846 -0.9262

tau^2 (estimated amount of residual heterogeneity): 0.0397 (SE = 0.0090)

tau (square root of estimated tau^2 value): 0.1993

I^2 (residual heterogeneity / unaccounted variability): 99.93%

H^2 (unaccounted variability / sampling variability): 1492.87

R^2 (amount of heterogeneity accounted for): 7.56%

Test for Residual Heterogeneity:

QE(df = 41) = 116184.4492, p-val < .0001

Test of Moderators (coefficients 2:5):

QM(df = 4) = 7.4684, p-val = 0.1131

Model Results:

estimate se zval pval ci.lb ci.ub

intrcpt 0.3250 0.1171 2.7742 0.0055 0.0954 0.5546 **

RegionsEastern Africa 0.0116 0.1255 0.0926 0.9262 -0.2344 0.2576

RegionsNorthern Africa 0.1863 0.1544 1.2061 0.2278 -0.1164 0.4890

RegionsSouthern Africa 0.2152 0.1472 1.4617 0.1438 -0.0734 0.5038

RegionsWestern Africa -0.0136 0.1290 -0.1051 0.9163 -0.2663 0.2392

---

Signif. codes: 0 ‘***’ 0.001 ‘**’ 0.01 ‘*’ 0.05 ‘.’ 0.1 ‘ ’ 1

*Figure 4. Meta-regression analysis of sepsis prevalence by country of study (no significant effect observed)*

*The following output summarizes the results from the mixed-effects meta-regression model using by country as a continuous moderator (method = REML).*

Mixed-Effects Model (k = 46; tau^2 estimator: REML)

logLik deviance AIC BIC AICc

3.1253 -6.2507 31.7493 57.0612 126.7493

tau^2 (estimated amount of residual heterogeneity): 0.0464 (SE = 0.0127)

tau (square root of estimated tau^2 value): 0.2154

I^2 (residual heterogeneity / unaccounted variability): 99.96%

H^2 (unaccounted variability / sampling variability): 2485.97

R^2 (amount of heterogeneity accounted for): 0.00%

Test for Residual Heterogeneity:

QE(df = 28) = 113367.0036, p-val < .0001

Test of Moderators (coefficients 2:18):

QM(df = 17) = 13.5159, p-val = 0.7010

Model Results:

estimate se zval pval ci.lb ci.ub

intrcpt 0.1859 0.2160 0.8606 0.3894 -0.2375 0.6093

PaysBotswana 0.0847 0.3054 0.2773 0.7815 -0.5139 0.6833

PaysCameroon 0.0343 0.3064 0.1119 0.9109 -0.5663 0.6349

PaysCentral African Republic 0.0802 0.3078 0.2605 0.7944 -0.5231 0.6835

PaysCongo 0.1684 0.2656 0.6339 0.5261 -0.3521 0.6888

PaysEgypt 0.3211 0.2498 1.2852 0.1987 -0.1685 0.8107

PaysEthiopia 0.1765 0.2258 0.7818 0.4344 -0.2660 0.6190

PaysGambia and Burkina Faso 0.3746 0.3078 1.2172 0.2235 -0.2286 0.9778

PaysGhana 0.0250 0.2497 0.1000 0.9203 -0.4644 0.5143

PaysKenya 0.0427 0.3055 0.1398 0.8888 -0.5560 0.6414

PaysMadagascar 0.2441 0.3064 0.7967 0.4256 -0.3564 0.8446

PaysMorocco 0.3378 0.3061 1.1034 0.2699 -0.2622 0.9378

PaysNigeria 0.1608 0.2294 0.7009 0.4834 -0.2888 0.6103

PaysRwanda -0.1361 0.3052 -0.4459 0.6557 -0.7343 0.4621

PaysSouth Africa 0.4213 0.2414 1.7456 0.0809 -0.0517 0.8944

PaysTanzania 0.1321 0.2648 0.4989 0.6178 -0.3868 0.6510

PaysUganda 0.1758 0.2500 0.7033 0.4819 -0.3141 0.6657

PaysZambia 0.1432 0.3062 0.4676 0.6401 -0.4570 0.7434

intrcpt

PaysBotswana

PaysCameroon

PaysCentral African Republic

PaysCongo

PaysEgypt

PaysEthiopia

PaysGambia and Burkina Faso

PaysGhana

PaysKenya

PaysMadagascar

PaysMorocco

PaysNigeria

PaysRwanda

PaysSouth Africa .

PaysTanzania

PaysUganda

PaysZambia

---

Signif. codes: 0 ‘***’ 0.001 ‘**’ 0.01 ‘*’ 0.05 ‘.’ 0.1 ‘ ’ 1

*Figure 5 : Meta-Regression of Sepsis Prevalence by Clinical Diagnostic Criteria study (no significant effect observed)*

*The following output summarizes the results from the mixed-effects meta-regression model using Clinical Diagnostic Criteria as a continuous moderator (method = REML)*

Mixed-Effects Model (k = 46; tau^2 estimator: REML)

logLik deviance AIC BIC AICc

5.3509 -10.7019 3.2981 15.1203 6.7981

tau^2 (estimated amount of residual heterogeneity): 0.0444 (SE = 0.0101)

tau (square root of estimated tau^2 value): 0.2108

I^2 (residual heterogeneity / unaccounted variability): 99.79%

H^2 (unaccounted variability / sampling variability): 465.62

R^2 (amount of heterogeneity accounted for): 0.00%

Test for Residual Heterogeneity:

QE(df = 40) = 108193.1962, p-val < .0001

Test of Moderators (coefficients 2:6):

QM(df = 5) = 3.4794, p-val = 0.6265

Model Results:

estimate se zval pval ci.lb

Clinical_definitions(EC)**^5^** 0.3894 0.0454 8.5672 <.0001 0.3003

Clinical_definitionsIPSC**^1^** -0.1050 0.1315 -0.7982 0.4248 -0.3628

Clinical_definitionsOther**^6^** -0.0381 0.0713 -0.5352 0.5925 -0.1778

Clinical_definitionsWHO_IMCI**^2^** -0.0789 0.1308 -0.6031 0.5464 -0.3352

Clinical_definitionsWHO_PSBI**^3^** 0.1536 0.1576 0.9749 0.3296 -0.1552

Clinical_definitionsWHO_YISG**^4^** -0.2473 0.2160 -1.1449 0.2523 -0.6705

ci.ub

Clinical_definitions(EC) 0.4784 ***

Clinical_definitionsIPSC 0.1528

Clinical_definitionsOther 0.1015

Clinical_definitionsWHO_IMCI 0.1774

Clinical_definitionsWHO_PSBI 0.4625

Clinical_definitionsWHO_YISG 0.1760

---

Signif. codes: 0 ‘***’ 0.001 ‘**’ 0.01 ‘*’ 0.05 ‘.’ 0.1 ‘ ’ 1

*.*

NB:

1 **Goldstein et al. (2005)** — International Pediatric Sepsis Consensus Conference

2 **IMCI (WHO 2005)** — Integrated Management of Childhood Illnesses

3 **WHO Guideline (2015)** — Possible Serious Bacterial Infection (PSBI)

4 **WHO Young Infant Study Group (1999)**

5 **Empirical_clinical**

6 **Other (considered as defined by the attending clinicians OR/Not specified)**


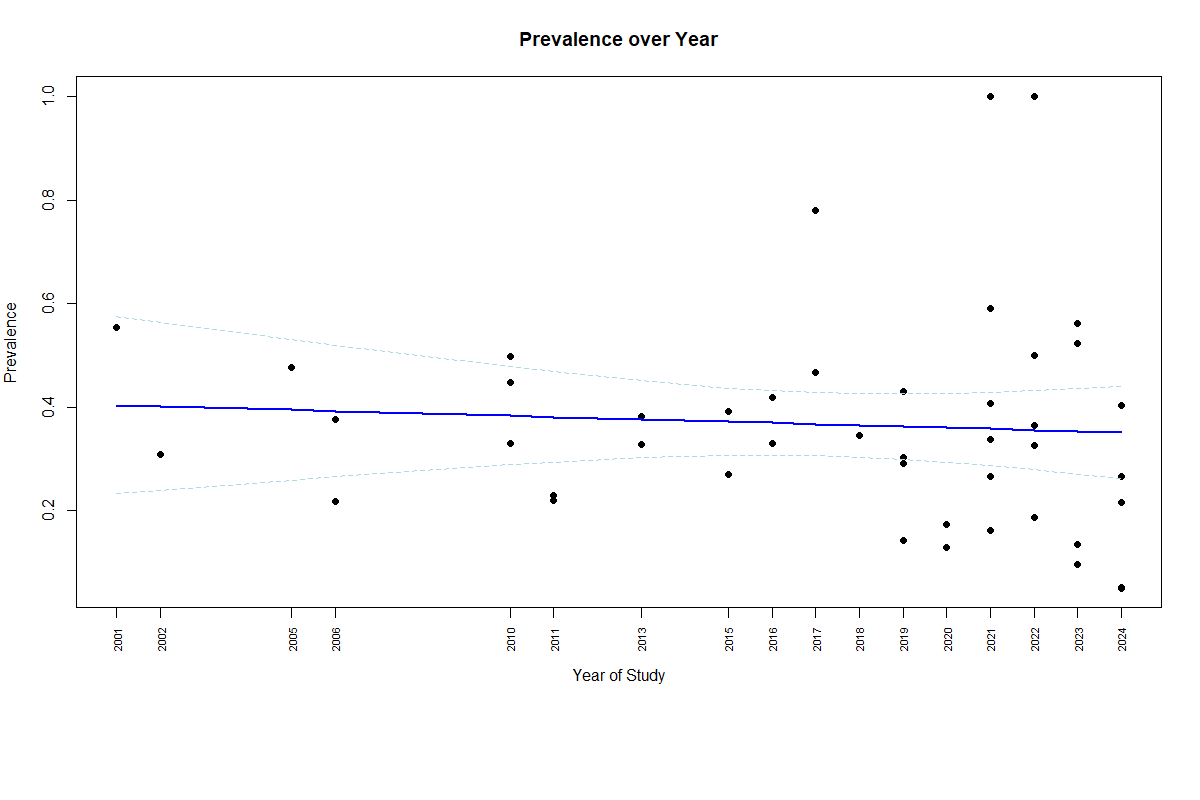
Figure 1 : Meta-regression plot assessing the association between year of publication and prevalence of sepsis (no significant effect observed)

Figure 2 : Meta-regression assessing the association between study design and prevalence of sepsis (no significant effect observed)


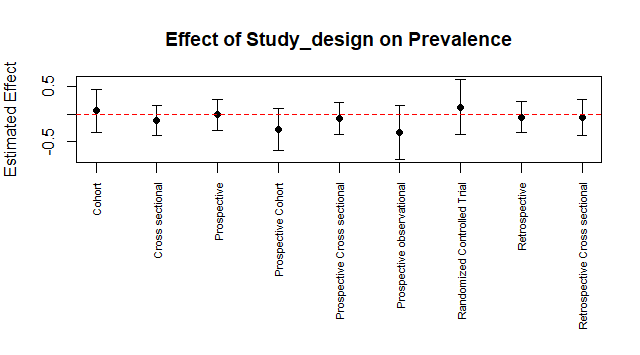


Figure 3 : Meta-regression plot evaluating the association between geographic region and prevalence of sepsis (no significant effect observed)


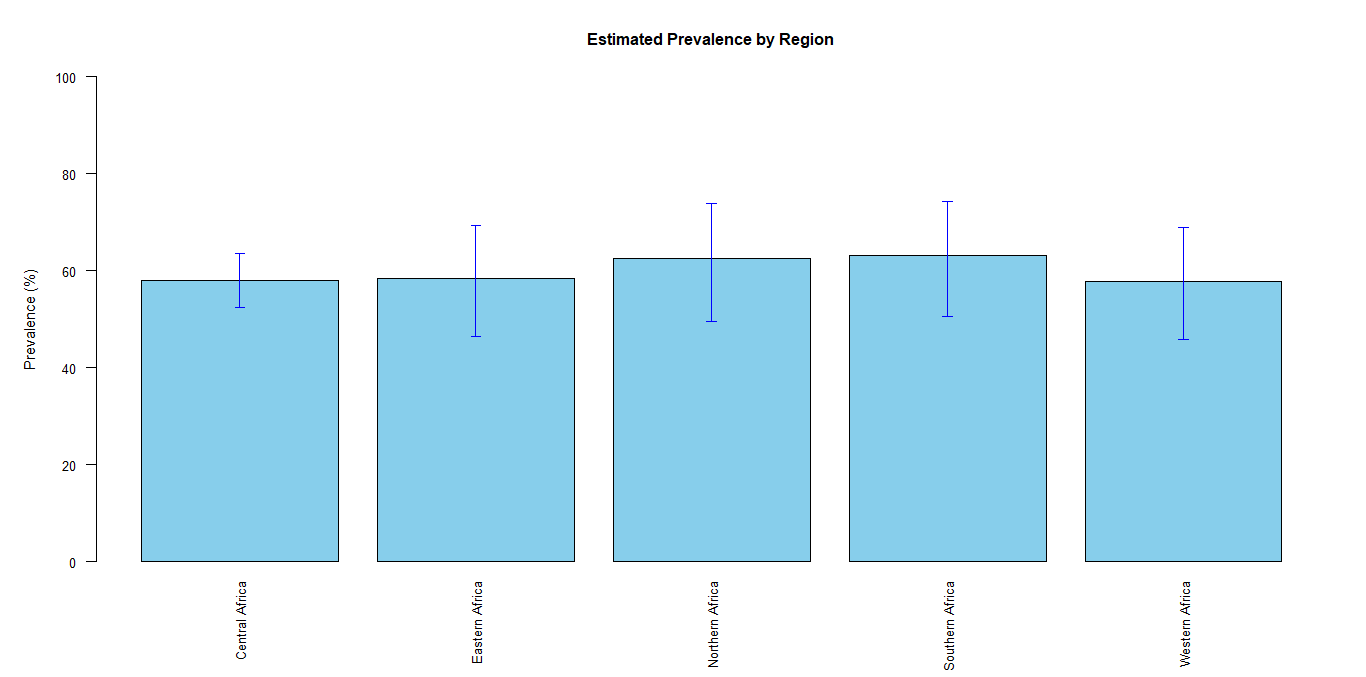


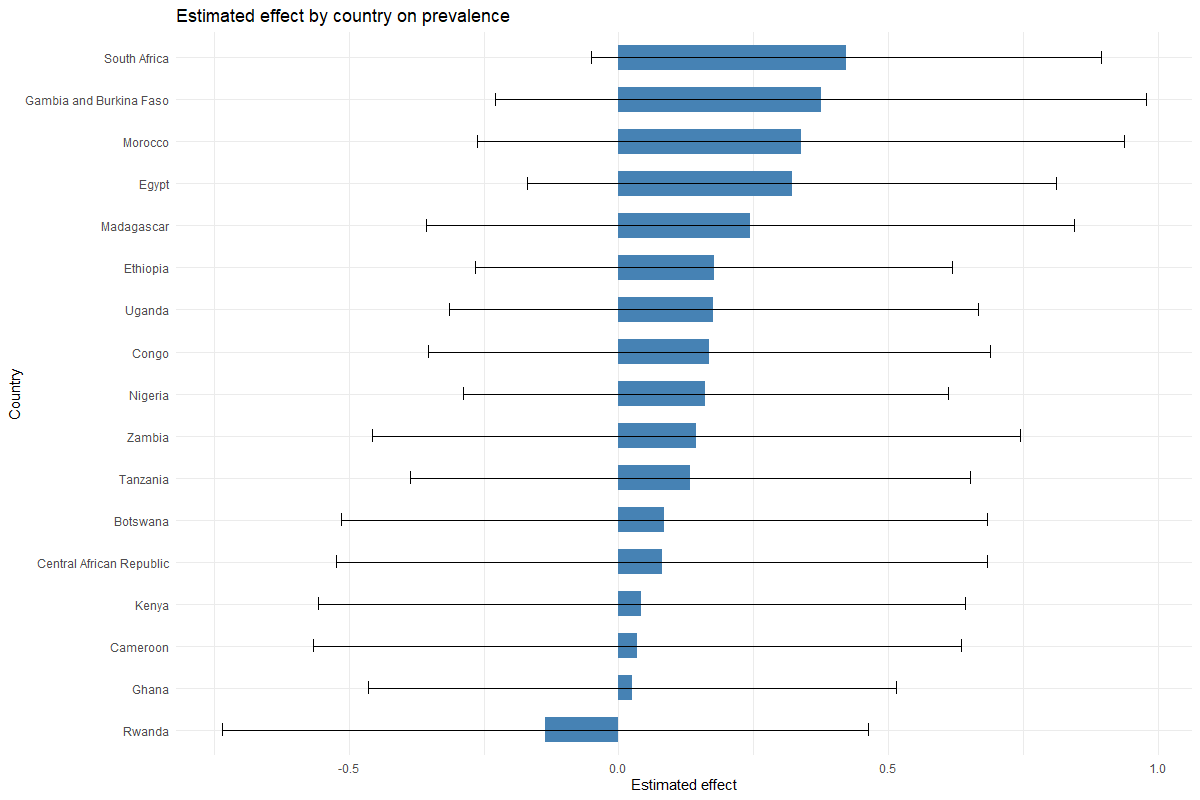


*Figure 4 Meta-regression analysis of sepsis prevalence by country of study (no significant effect observed)*


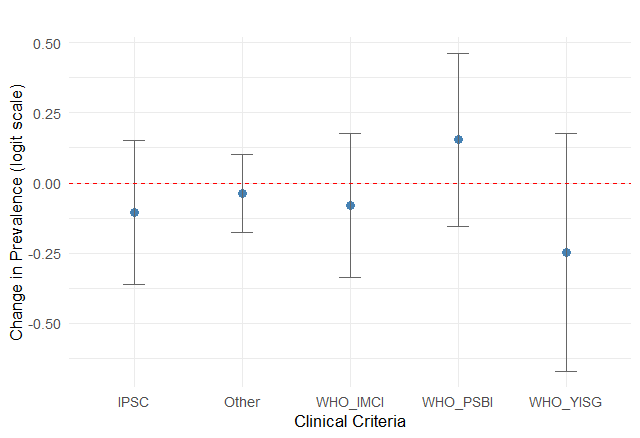


Figure 5 Meta-Regression analysis of Sepsis Prevalence by Clinical Diagnostic Criteria
